# Insulin alleviates murine colitis through microbiome alterations and bile acid metabolism

**DOI:** 10.1186/s12967-023-04214-3

**Published:** 2023-07-25

**Authors:** Shuying He, Jiating Li, Zirong Yao, Zixian Gao, Yonghong Jiang, Xueqing Chen, Liang Peng

**Affiliations:** Department of Gastroenterology, First Affiliated Hospital of Guangzhou Medical University, Guangzhou Medical University, No. 151, Yanjiang West Road, Yuexiu District, Guangzhou, 510120 Guangdong People’s Republic of China

**Keywords:** Insulin, Gut microbiota, Lithocholic acid, Inflammatory bowel disease, Macrophage

## Abstract

**Background:**

Insulin has been reported to exhibit anti-inflammatory activities in the context of bowel inflammation. However, the role of the interaction between insulin and the microbiota in gut health is unclear. Our goal was to investigate the mechanism of action of insulin in bowel inflammation and the relationship between insulin and the gut microbiota.

**Methods:**

We used acute and chronic murine models of inflammatory bowel disease (IBD) to evaluate whether insulin influences the progression of colitis. Colonic tissues, the host metabolome and the gut microbiome were analyzed to investigate the relationship among insulin treatment, the microbiome, and disease. Experiments involving antibiotic (Abx) treatment and fecal microbiota transplantation (FMT) confirmed the association among the gut microbiota, insulin and IBD. In a series of experiments, we further defined the mechanisms underlying the anti-inflammatory effects of insulin.

**Results:**

We found that low-dose insulin treatment alleviated intestinal inflammation but did not cause death. These effects were dependent on the gut microbiota, as confirmed by experiments involving Abx treatment and FMT. Using untargeted metabolomic profiling and 16S rRNA sequencing, we discovered that the level of the secondary bile acid lithocholic acid (LCA) was notably increased and the LCA levels were significantly associated with the abundance of *Blautia*, *Enterorhadus* and *Rumi-NK4A214_group*. Furthermore, LCA exerted anti-inflammatory effects by activating a G-protein-coupled bile acid receptor (TGR5), which inhibited the polarization of classically activated (M1) macrophages.

**Conclusion:**

Together, these data suggest that insulin alters the gut microbiota and affects LCA production, ultimately delaying the progression of IBD.

**Graphical Abstract:**

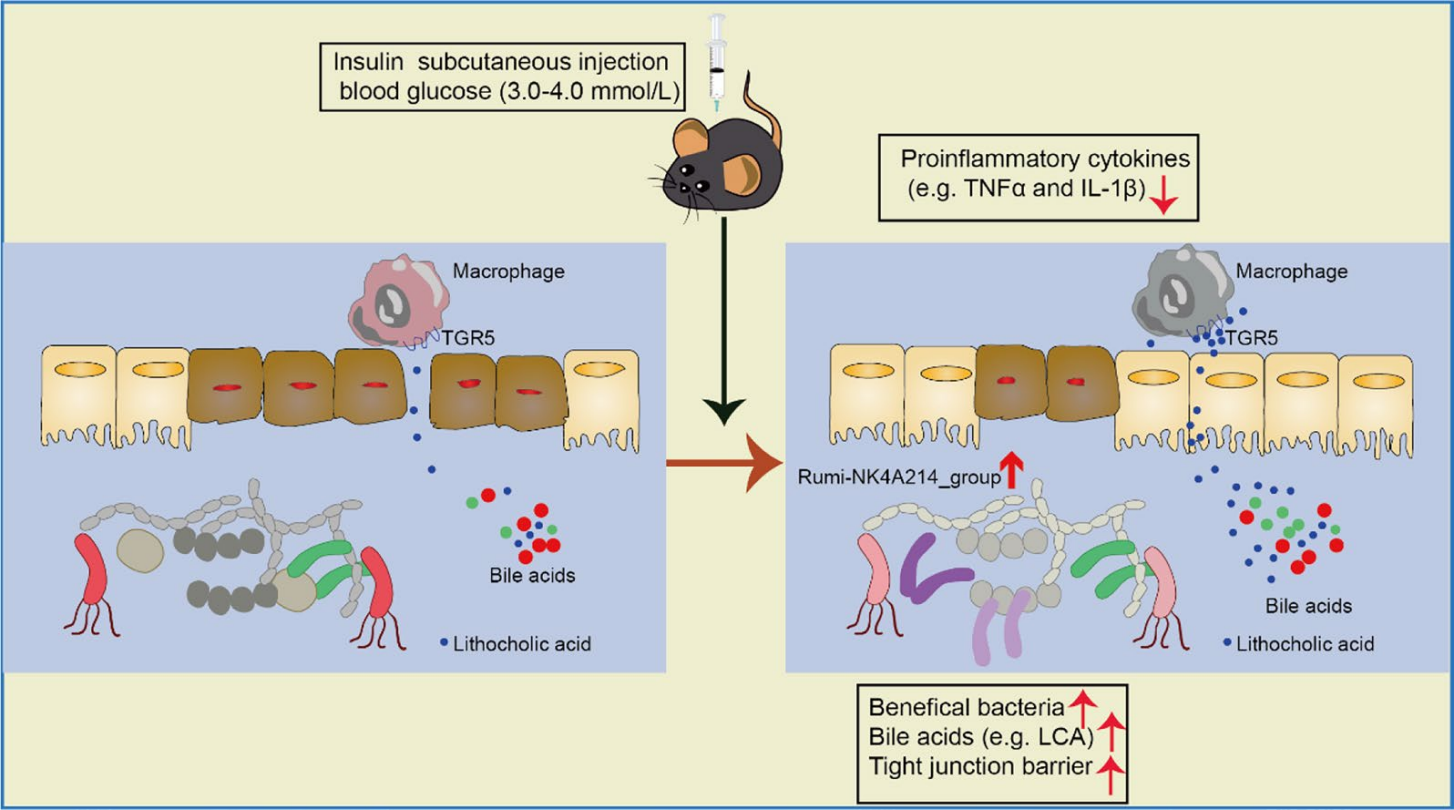

**Supplementary Information:**

The online version contains supplementary material available at 10.1186/s12967-023-04214-3.

## Introduction

Inflammatory bowel disease (IBD), including ulcerative colitis (UC) and Crohn’s disease (CD), and the incidence and prevalence of these two clinical phenotypes are increasing worldwide [1–3]. IBD patients usually undergo chronic and relapsing inflammation in the gastrointestinal tract [4]. Corticosteroids, immunosuppressants and biologicals hold great promise to provide the most beneficial therapy to improve the IBD course of care for an individual patient [5]. Nevertheless, these drugs are associated with substantial adverse events [6]. Recently, studies have found that insulin, the only pharmacotherapy that can directly lower blood glucose levels through stimulation of glucose uptake in muscle and fat tissues [7, 8], can have anti-inflammatory, antioxidant, and antithrombotic effects in patients with acute myocardial infarction independent of a decrease in glucose concentrations [9–11], indicating that insulin could be applied in other inflammatory conditions, including IBD. Moreover, studies have shown that insulin alleviates inflammation in a murine colitis model by being instilled rectally [12]. These results provide clues for the existence of crosstalk between insulin and inflammation in the bowel. However, the impact of such crosstalk on IBD remains to be further explored.

Dysbiosis is considered a causal or synergistic factor in IBD [13–18], and fecal microbiota transplantation (FMT) has already emerged as a successful therapy for IBD [17, 19–21]. FMT can introduce specific bacteria or probiotics to shift the composition to a healthier state [17]; in addition, the gut microbiome also produces a variety of metabolites, and manipulating the intestinal microbiota represents a potential treatment for IBD [22]. For example, the generation of secondary bile acids (SBAs) relies on two key bacteria-related processes, bile acid deconjugation and 7a-hydroxylation [23, 24], which in turn influence the physiologic response in the intestine differently [23, 25]. To our surprise, some studies also showed that insulin can modulate the microbiota, and the baseline microbiota can positively and negatively affect the pharmacodynamics [8, 26, 27]. Another oral antidiabetic drug, that is, metformin, alleviates intestinal inflammation by restoring the integrity of the intestinal epithelial barrier and altering the gut microbiome composition [28–31]. It is therefore of interest to broaden our understanding of the potential link between insulin and gut microbiota in IBD.

In our study, we investigated the mitigating effects of insulin on inflammation in both acute and chronic murine colitis models and the expression of key cytokines involved in macrophage recruitment. We further studied the microbiota and metabolite differences in the feces of insulin-treated or untreated acute colitis mice. In our metabolomic studies, the SBA lithocholic acid (LCA), which is known to reduce the inflammatory response [25, 32], was present in significantly higher amounts in the feces from the insulin-treated group than in the feces from the untreated group. Importantly, 16S rRNA gene sequencing studies showed that the abundance of *Blautia*, *Enterorhadus* and *Rumi-NK4A214_group*, which are capable of generating SBAs [33, 34], was significantly associated with the concentration of LCA. Finally, we investigated the effects of LCA on anti-inflammatory and classically activated macrophage (M1) polarization and the roles of G-protein-coupled receptor 5 (TGR5, also known as Gpbar1) in LCA-mediated M1 macrophage activation. This study revealed a critical role for insulin in alleviating inflammation and suggests potential pharmaceutical applications in IBD.

## Materials and methods

### Blood glucose measurement in mice

For insulin treatment, insulin (0.01 U in 100 μL of phosphate-buffered saline (PBS) per mouse, Sigma–Aldrich Corp., St. Louis, MO, USA) was administered via intraperitoneal injection. The glucose levels in tail vein blood samples were measured at 0, 15, 30, 60, 90, 120, 180, 240, 300 and 360 min after insulin injection. The blood glucose concentration was measured by using a commercially available glucose assay kit (Cell Biolabs, Inc., CA, USA).

### Mouse husbandry and models

Wild-type (WT) C57BL/6 mice (6–8 weeks old) were purchased from Guangdong Medical Laboratory Animal Center (Guangzhou, China). All animal protocols were approved by the Animal Ethics Committee of Guangzhou Medical University. The animals were maintained under specific pathogen-free conditions in the Guangzhou Medical University Animal Experiment Center (Guangzhou, China). For the in vivo experiment, disease activity index (DAI) was determined as we previously reported [35]. Body weight changes were monitored daily. Colon tissues were harvested, and the colon length was measured. Serum was collected and used for Luminex assays. Fecal material was collected at the time of sacrifice, snap frozen and stored at -80℃ for metabolomic and 16S rRNA sequencing analyses and fecal microbiota transplantation (FMT).

To elucidate the effect of insulin on the colon, the mice were treated with insulin (0.01 U/mouse) or PBS alone (n = 10 in each group) on days 1, 3 and 5, and then colon tissues were harvested on day 6. Short-acting insulin (recombinant human insulin) was obtained from Sigma-Aldrich (St. Louis MO, USA).

To establish a model of DSS-induced colitis, mice were administered 2.5% DSS (MW 40000–50000, MP Biomedicals, Solon, Ohio, USA) in distilled water for 5 days and then given normal drinking water. The mice were injected with PBS (n = 7) or insulin (n = 9) intraperitoneally on day 3, day 5 and day 7. To establish a model of DSS-induced chronic colitis, the cycle of DSS-containing water and normal water administration was repeated three times, and the mice were also treated with PBS (n = 9) or insulin (n = 11). For LCA treatment, solutions of LCA (Sigma–Aldrich) were prepared by mixing with ultrapure water and then sonicating. The volume for all enemas was 100 μL, and all enema suspensions contained either 5 mg of LCA or ultrapure water. The enema suspensions were instilled via the rectum using a 1-mL syringe on days 3, 5 and 7 (n = 8 in each group).

To establish a model of TNBS-induced colitis, mice were presensitized for 1 week and anesthetized with sevoflurane. Then, the mice were administered TNBS/45% ethanol (2.5% TNBS in 100 μL of ethanol) via a 3.5-F catheter equipped with a 1-mL syringe, which was inserted into the colon until the tip was 4 cm proximal to the anal verge; then, 100 μL of TNBS was infused [36]. Insulin was administered intraperitoneally on days 1, 2 and 3 (n = 7 in each group).

For glycolysis inhibition, mice were pretreated with 2-DG (1 g/kg) (Sigma–Aldrich) intraperitoneally before the administration of insulin (n = 6 in each group).

To treat the mice with Abx, a well-established cocktail of four broad-spectrum Abxs (1 g/L neomycin, 1 g/L metronidazole, 1 g/L ampicillin, and 0.5 g/L vancomycin) (Sigma–Aldrich) was added to the drinking water for 5 days. Then, the water was changed to 2.5% DSS, and the mice were treated with insulin (n = 7 per group).

FMT was performed according to the protocol reported in our previous study. After 5 days of Abx treatment, mice were administered 2.5% DSS and inoculated with fresh transplant material (20 mg of feces/100 µl of PBS) or PBS by oral gavage every day for the whole experimental period (n = 7 per group). Feces were collected from PBS-treated mice or insulin-or PBS-treated mice with DSS-induced colitis.

GW4064 (Selleck Chemicals LLC, USA) was orally administered at a dose of 10 mg/kg, and two doses were given at a 12 h interval. Then, 2.5% DSS was given, and insulin and LCA were administered as described above.

To generate a TGR5 knockdown model, mice were intraperitoneally injected with methylated siRNA (GenePharma, Shanghai, China) targeting TGR5 (1 OD/mouse) on days -3, 0 and 2 of the experiment. Three days after the first dose of siRNA, 2.5% DSS was given, and insulin and LCA were administered as described above. The sequences of the negative control and si-*TGR5* are presented in Additional file 1: Table S1.

### Histopathology and immunohistochemistry (IHC)

Colon tissues were washed with PBS, fixed in 4% paraformaldehyde and embedded in paraffin. Tissue sections of the whole colon were subjected to hematoxylin & eosin (H&E) and periodic acid Schiff (PAS) staining (Solarbio, Beijing, China). These procedures were performed according to the manufacturer’s protocols. The stained sections were imaged under a Leica DM6 microscope (Leica, Wetzlar, Germany). Histological scoring was performed in a blinded fashion by two pathologists. Colitis was scored based on the combined scores of inflammatory cell infiltration (score 0–4), ulceration (score 0–4) and area of crypt distortion (score 0–4).

For IHC, 4% paraformaldehyde-fixed and paraffin-embedded colon tissue sections were deparaffinized and hydrated with decreasing concentrations of ethanol. Tissue sections were stained for Ki67 using mouse anti-Ki67 antibodies (ab279653), F4/80 using rabbit anti-F4/80 antibodies (ab16911) and iNOS using rabbit anti-iNOS antibodies (ab3523); the antibodies were obtained from Abcam (Cambridge, MA, USA). Images were taken using a Leica DM6 microscope (Leica). The investigators examined the lesions in a blinded fashion.

### Quantitative real-time PCR

Total RNA was extracted with TRIzol reagent (Thermo Fisher Scientific, Waltham, MA, USA), and cDNA was synthesized using PrimeScriptTM RT Master Mix (Takara, Dalian, China). Quantitative real-time PCR was performed with the primers listed in Additional file 1: Table S1 using SYBR Premix Ex Taq (Vazyme, Nanjing, China) and the Bio-Rad CFX96 System (Bio–Rad Laboratories, CA, USA). The expression of glycolysis-associated genes in the samples was normalized to the expression of hypoxanthine phosphoribosyltransferase (Hprt), while the expression of other genes was normalized to the expression of the housekeeping gene glyceraldehyde 3-phosphate dehydrogenase (Gapdh). Relative mRNA expression was calculated using the comparative 2^−△△CT^ method. Fecal genomic DNA was extracted using the QiaAmp DNA Mini Kit (Qiagen, Valencia, CA, USA) according to the manufacturer’s instructions. To normalize the biopsy size, the concentration of total bacteria (Eubacteria) from mice was standardized based on 16S rRNA levels.

### Western blot analysis

For western blot analyses, total protein samples were isolated from tissues with RIPA lysis buffer supplemented with a protease inhibitor cocktail (Roche, Basel, Switzerland) and phosphatase inhibitor (Roche). An anti-TGR5 primary antibody (ab72608) was purchased from Abcam, and an anti-GAPDH primary antibody was purchased from Zhonshanjinqiao (Wuhan, China). The Gene5 image acquisition system (Syngene, Frederick, MD, USA) was used for signal detection.

### 16S rRNA gene analysis

Mouse fecal bacterial DNA extraction, 16S rRNA gene PCR amplification and sequencing and 16S rRNA gene analysis were performed by Gene Denovo Biotechnology Company (Guangzhou, China). Fecal bacterial DNA was extracted from each sample, and the integrity and size of the DNA samples were determined by 1% agarose gel electrophoresis. The primers 341F (CCTACGGGNGGCWGCAG) and 806R (GGACTACHVGGGTATCTAAT) were used to amplify the V3–V4 region of the 16S rRNA gene. The 16S rRNA gene sequences were analyzed using operational taxonomic units (OTUs) selected with a threshold of 97% pairwise identity and classified taxonomically using the Ribosomal Database Project (RDP) classifier 2.0.1.

### Untargeted metabolomics

Metabolomics analyses of stool samples were performed using a TripleTOF_5600_plus system (SCIEX, UK) with a high‐resolution tandem mass spectrometer as described previously [25]. Briefly, fecal samples were suspended in methanol (5 μL/mg stool), vortexed for 1 min, and centrifuged at 13,000×*g* for 5 min. The supernatants were transferred to new vials and analyzed on an Acquity UPLC T3 column (100 mm*2.1 mm, 1.8 µm, Waters, UK). All the chemicals and reagents were mass spectrometry grade. Differential metabolite level analysis was performed using the two-sided Wilcoxon signed-rank test. Untargeted metabolomic analysis was performed in collaboration with LC Sciences, LLC (Hangzhou, China).

### Targeted metabolomics

Targeted metabolomics analysis of bile acids from mouse stool was performed at LC Sciences, LLC. Briefly, fecal samples were mixed with acetonitrile-methanol (8:2 v/v). The supernatant obtained during extraction was used for UPLC–MS analysis. An aliquot of stock solution was prepared by mixing bile acid standards. A Waters Acquity ultra performance LC system equipped with a binary solvent delivery manager and a sample manager (Waters, Milford, MA) was used for bile acid (BA) analysis. The mass spectrometer was a Waters XEVO TQ instrument with an ESI source (Waters Corp., Milford, MA). The entire LC–MS system was controlled by MassLynx 4.1 software [37].

### Quantification of cytokine levels with Luminex

A mouse high-sensitivity T-cell magnetic bead panel was obtained from Merck Millipore (Darmstadt, Germany), and the experiment was conducted according to the manufacturer’s instructions. All samples were measured in duplicate. Standard curves of known concentrations of recombinant human cytokines/chemokines were used to convert fluorescence units to cytokine concentration units (pg/mL). All results below the minimum concentrations were processed as the minimum concentrations.

### Preparation of bone marrow-derived macrophages (BMDMs)

Isolated bone marrow (BM) cells were used as previously reported [38]. The cells were cultured in medium supplemented with 10% FBS and GM-CSF (10 ng/mL, PeproTech, Rocky Hill, NJ). On day 7, the cells were further treated with 20 ng/mL IFNγ (PeproTech) plus 20 ng/mL LPS (PeproTech). For si-TGR5 transfection, BMDMs from WT mice were transfected with si-TGR5 or si-NC for 12 h, and the cells were then stimulated with IFNγ plus LPS for 4 h or 24 h. For the LCA treatment, BMDMs were treated with LCA (50 μM) for 12 h and then stimulated with IFNγ plus LPS for 4 h or 24 h. For treatment, the sterile supernatants of cecal contents were prepared by homogenizing cecal content in PBS (10% w/v) before centrifugation (12,000*g*, 10 min, 4 °C) and filtration (0.22 µM) in an aseptic environment and diluted in cell culture medium (10% v/v). The cell supernatant was collected for ELISA, and the cells were harvested for qPCR and flow cytometry analyses. The samples were tested in triplicate.

### ELISA

The concentrations of TNF-α and IL12p40 in the cell supernatants were determined using an ELISA kit (4A Biotech, Beijing, China) according to the manufacturer’s instructions. The samples were tested in triplicate.

### Isolation of lymphocytes from the intestine

Lymphocytes from the intestine were isolated as previously described [39]. These lymphocytes were used for flow cytometry analysis.

### Flow cytometry analysis

Lymphocytes were isolated from the intestines of mice. The cells were stained with fluorescence-conjugated antibodies from eBioscience (San Diego, CA, USA) or BioLegend (San Diego, CA, USA) (Additional file 1: Table S2). For intracellular transcription factor staining, the cells were stimulated with Cell Stimulation Cocktail (Plus Protein Transport Inhibitors) (eBioscience) for 4 h and stained using a Cytofix/Cytoperm staining kit (BD Biosciences, San Diego, CA, USA). Flow cytometry was performed on a FACSVerse cytometer (BD Biosciences, San Jose, CA, USA), and the data were subsequently analyzed using FlowJo 10.0 (Tree Star, Ashland, OR, USA).

### Statistical analysis

GraphPad Prism 8.0 software (GraphPad Software Inc., USA) was used for data analysis. Statistical analysis was performed using Student’s t test, and comparisons of more than two groups were performed by one-way ANOVA. *P* < 0.05 was used to indicate statistical significance.

## Results

### Intraperitoneal insulin mitigates intestinal inflammation in murine colitis

**Fig. 1:**
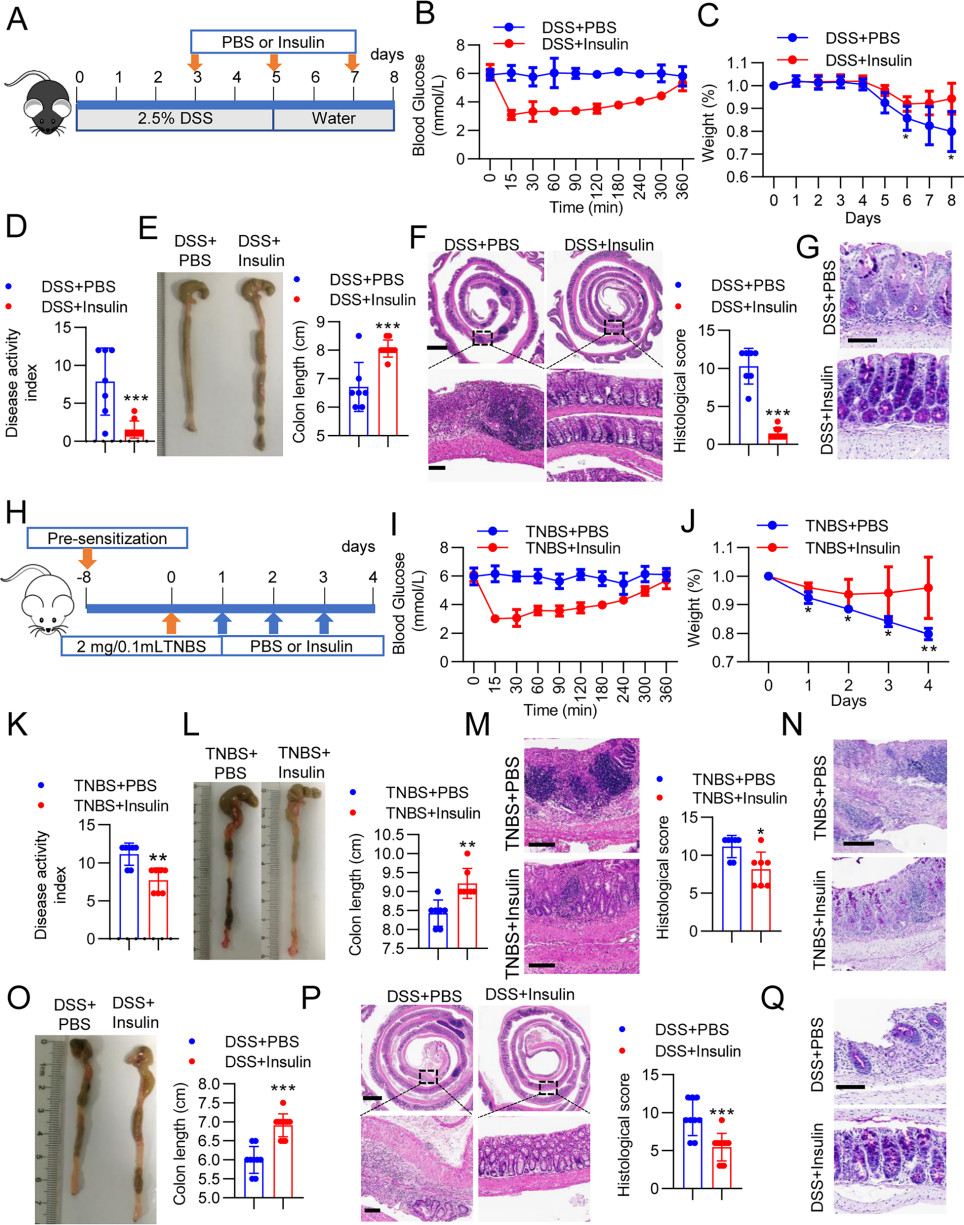
Insulin ameliorated colitis in the murine model. **A**–**G** WT mice were treated with DSS for 5 days, followed by 3 days of normal drinking water, and the mice were treated with PBS (n = 7) or insulin (n = 9) intraperitoneally on day 3, day 5 and day 7. **B** Blood glucose levels were measured on day 3. **C** Body weight was monitored daily after DSS administration. **D** DAI scores and **E** colon length in PBS- and insulin-treated mice with DSS-induced colitis. **F** Paraffin-embedded sections of PBS- and insulin-treated mouse colons were analyzed by H&E. Upper scale bar, 200 μm; lower scale bar, 100 μm. **G** Paraffin-embedded sections of PBS- and insulin-treated mouse colons were analyzed by PAS. Scale bar, 100 μm. **H**–**M** Mice were presensitized with topical TNBS, further sensitized via recto-transfer of TNBS, and treated with PBS or insulin on day 1, day 2 and day 3 (n = 7 in each group). **I** Blood glucose levels were measured on day 1. **J** Body weight was monitored daily after TNBS administration. **K** DAI scores and **L** colon length in PBS- and insulin-treated mice administered TNBS were measured and histologically analyzed after H&E staining. **M** Scale bar, 100 μm. **N** Paraffin-embedded sections of PBS- and insulin-treated mouse colons were analyzed by PAS. Scale bar, 100 μm. **O** Insulin ameliorated chronic colitis. Mice were treated with DSS and insulin, and the cycle was repeated three times (PBS = 9, insulin = 11). Colon length in PBS- and insulin-treated chronic colitis mouse models. **P** Paraffin-embedded sections of PBS- and insulin-treated mouse colons were analyzed by H&E. Scale bar, 50 μm. **Q** Paraffin-embedded sections of PBS- and insulin-treated mouse colons were analyzed by PAS staining. Scale bar, 100 μm. The data represent the mean ± SD. ***P < 0.001. The data represent the mean ± SD. *P < 0.05; **P < 0.01; ***P < 0.001

Previous research has shown that insulin inhibits intestinal inflammation by enema [12]. However, insulin is usually injected subcutaneously. Therefore, we wanted to investigate whether intraperitoneal insulin also has a direct impact on intestinal inflammation. To test this hypothesis, colitis was induced by administering 2.5% dextran sulfate sodium (DSS) in the drinking water over a 5-day period, and insulin (0.01 U/mouse) was administered via intraperitoneal injection on days 3, 5 and 7 (Fig. 1A). We first assessed the blood glucose concentrations affected by insulin. The glucose nadir (3.1 ± 0.3 mmol/L) was observed at 15 min and continued to 30 min (3.3 ± 0.7 mmol/L) after insulin treatment. Then, after insulin treatment, the glucose concentrations slowly increased to reach values (5.3 ± 0.5 mmol/L) comparable to those seen after PBS treatment (5.8 ± 0.7 mmol/L) at 360 min (Fig. 1B). On days 5 and 7, similar changes in blood glucose concentrations were recorded (Additional file 1: Fig. S1A). Notably, we did not observe the death of any mice after treatment with insulin.

Next, we characterized the effect of insulin on DSS-induced colitis. Insulin treatment maintained body weight with higher efficacy than PBS control treatment (Fig. 1C). Consistently, insulin intervention significantly alleviated DSS-induced colitis, as evidenced by markedly reduced DAI scores, which were calculated by measuring body weight loss, stool consistency, and blood in the stool, and relieved colonic shortening (Fig. 1D, E). Histological assessments showed obvious attenuation of inflammatory cell infiltration and mucosal damage and reduced overall histological scores for the colon in response to insulin treatment (Fig. 1F and Additional file 1: Fig. S2). Similarly, the inflammatory response in insulin-treated mice was ameliorated, as indicated by significantly decreased expression of *interleukin (Il) 6*, *Il1β* and *tumor necrosis factor (Tnf)α* (Additional file 1: Fig. S1B). To assess the effect of insulin on the colonic mucosal barrier, mucin-secreting goblet cells in the colonic epithelium were evaluated by PAS staining, and insulin injection increased the number of goblet cells (Fig. 1G). Furthermore, insulin had an obvious effect on promoting the transcript levels of the tight junction proteins *zonula occludens 1* (*Zo-1*) and *occludin*, which were drastically elevated in the colonic mucosa of insulin-treated mice (Additional file 1: Fig. S1C).

To further confirm the findings from the DSS-induced acute colitis model, we also conducted experiments assessing whether insulin reduces intestinal inflammation in other acute and chronic colitis models. We first examined the ability of insulin to alleviate acute colitis in the 2,4,6-trinitrobenzenesulfonic acid solution (TNBS)-induced acute colitis model (Fig. 1H). The glucose nadir was again observed at 15 min, and the blood glucose concentration slowly increased to normal levels at 360 min (Fig. 1I and Additional file 1: Fig. S1D). Furthermore, insulin-treated mice showed decreased colitis susceptibility accompanied by less body weight loss and clinical signs of colitis than insulin-untreated controls (Fig. 1J, K). By day 4, the colon of insulin-treated mice was significantly longer and showed reduced inflammatory cell infiltration, crypt loss, and ulceration (Fig. 1L, M). The number of mucus-filled goblet cells was also increased in the colon after insulin treatment (Fig. 1N). Accordingly, the expression of proinflammatory factors (*Il6*, *Il1β* and *Tnfα*) was decreased, and that of barrier proteins (*Zo-1* and *occludin*) was significantly increased in colon tissues from mice treated with insulin (Additional file 1: Fig. S1E-F). Next, we tested the effect of insulin in the chronic DSS-induced colitis model. The DSS treatment cycle was repeated three times. Insulin reproducibly reduced colitis, as shown by analysis of the colon length, histological assessments and evaluation of the goblet cells (Fig. 1O–Q). These data demonstrate that intraperitoneal insulin also alleviates acute and chronic murine colitis.

### Low-dose insulin mitigates intestinal inflammation independent of glycolysis

**Fig. 2:**
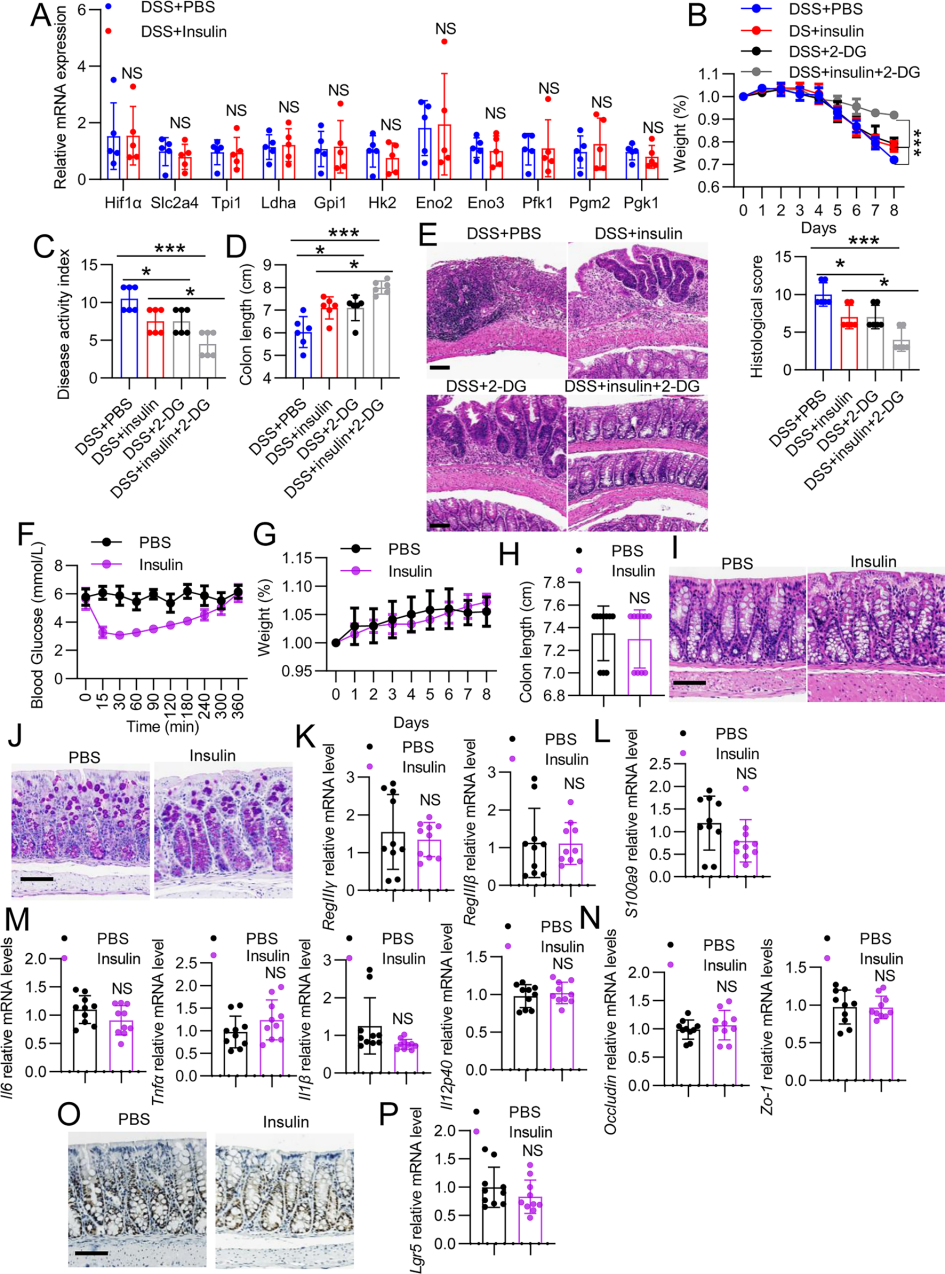
Low-dose insulin does not affect glycolysis and gut physiology. **A** WT mice were treated with DSS for 5 days and then were treated with insulin (n = 6 in each group). qPCR analysis of glycolysis-associated genes in murine colon tissues. **B**–**E** Mice were pretreated with 2-DG (1 g/kg) (Sigma–Aldrich) intraperitoneally before the administration of insulin (n = 6 in each group). **B** Body weight changes were monitored daily after DSS administration. **C** DAI scores and **D** colon length in all the groups. **E** Representative images of H&E staining of colon sections from different treatments. **F**–**P** Mice were treated with insulin or PBS alone (n = 10 in each group) on days 1, 3 and 5, and then colon tissues were harvested on day 6. **F** Blood glucose was determined on day 1. **G** Body weight was monitored daily. **H** Colon length in PBS- and insulin-treated mice. **I** Paraffin-embedded sections of PBS- and insulin-treated mouse colons were analyzed by H&E. Scale bar, 100 μm. **J** Paraffin-embedded sections of PBS- and insulin-treated mouse colons were analyzed by PAS. Scale bar, 100 μm. **K**–**M** mRNA expression levels of the indicated genes in the colonic mucosa of PBS- and insulin-treated mice. **O** Immunohistochemical staining of Ki67 in PBS- and insulin-treated mouse colons. Scale bar, 50 μm. **P** mRNA expression levels of *Lgr5* in the colonic mucosa of PBS- and insulin-treated mice. The data represent the mean ± SD. NS, not significant

The presence of mucosal hypoxia during intestinal inflammation is evident, and it enhances glycolysis in intestinal inflammation [4]; moreover, the glycolytic inhibitor 2-deoxy-D-glucose (2-DG) can attenuate the inflammatory response in a mouse model of DSS [40]. Since the glucose levels were decreased after low-dose treatment, we sought to determine whether the glycolytic pathway impacted the anti-inflammatory effect during insulin treatment. First, we assessed the oxygen-sensitive transcription factor hypoxia-inducible factor (HIF) 1α. Referencing the DSS model shown in Fig. 1A, the expression of *Hif1α* was not affected by insulin (Fig. 2A). The glucose transporter solute carrier family 2 member 4 (Slc2a4) plays an important role in insulin-regulated glucose uptake [41]. We therefore asked whether insulin inhibited Slc2a4 to facilitate glucose uptake. Our results showed that *Slc2a4* remained at the steady state (Fig. 2A). Next, we also assessed glycolysis-associated key enzymes, including hexokinase 2 (*Hk2*), lactate dehydrogenase a (*Ldha*), triosephosphate isomerase 1 (*Tpi1*), phosphate isomerase 1 (*Gpi1*), enolase 2 (*Eno2*), *Eno3*, phosphofructokinase 1(*Pfk1*), phosphohexose 1 (*Pgm1*) and phosphoglycerate kinase 1 (*Pgk1*), and no significant differences were observed between the two groups (Fig. 2A). To validate this further, we treated mice with 2-DG and then challenged them with insulin. Treatment with insulin or 2-DG alleviated bowel inflammation as indicated by the body weight, colon length, DAI scores, and histological damage compared with those of the DSS + PBS group (Fig. 2B–E). However, insulin-treated mice that received 2-DG further showed significantly decreased DAI scores (Fig. 2C) and histological damage (Fig. 2E) as well as increased body weight (Fig. 2B) and colon length (Fig. 2D) compared with those of the other group, suggesting that the low-dose insulin-mediated anti-inflammatory response in colitis is independent of its effects on glycolysis.

### Short-term insulin treatment does not influence gut physiology in mice

Insulin-treated mice showed alleviated colitis development, leading us to consider whether short-term treatment with insulin affects the gut physiology of normal mice. We treated mice with insulin on days 3, 5, and 7. The change in the blood glucose concentration was consistent with the phenomena described in Fig. 1 (Fig. 2F). On day 8, we evaluated body weight, colon length and histopathology. No differences in body weight alterations or colon lengths were found between PBS- and insulin-treated mice (Fig. 2G, H). Histological analysis showed a normal epithelial architecture (Fig. 2I). Goblet cells, C-type lectin regenerating protein 3γ (REG3γ) and REG3β were also not changed by short-term exposure to insulin (Fig. 2J, K). Furthermore, S100 calcium-binding protein A9 (S100A9) expression as well as the induction of neutrophil chemotaxis and adhesion in the colon were comparable between the PBS- and insulin-treated groups [42] (Fig. 2L). Consistently, insulin- and PBS-treated colon tissues exhibited similar levels of proinflammatory factors (*Il6*, *Il1β*, *Tnfα*) and *Il12p40*) (Fig. 2M). Injury to epithelial cells is a colitis-initiating event that can increase epithelial barrier permeability; therefore, we examined the expression levels of *Zo-1* and *occludin*, which were not affected by insulin treatment (Fig. 2N). Defects in epithelial cell proliferation also play key roles in colitis pathogenesis and regeneration, but we did not observe effects of insulin on the proliferation of intestinal epithelial cells by Ki67 staining (Fig. 2O). Then, we performed qPCR analysis and found that the expression of leucine-rich repeat-containing receptor 5 (Lgr5), which is a marker of stem cells, was not affected by insulin intervention (Fig. 2P). Considering these observations, it appears that insulin does not induce apparent pathophysiological changes in the normal colonic mucosa.

### Insulin-mediated colitis pathogenesis alleviation is gut microbiota dependent

**Fig. 3:**
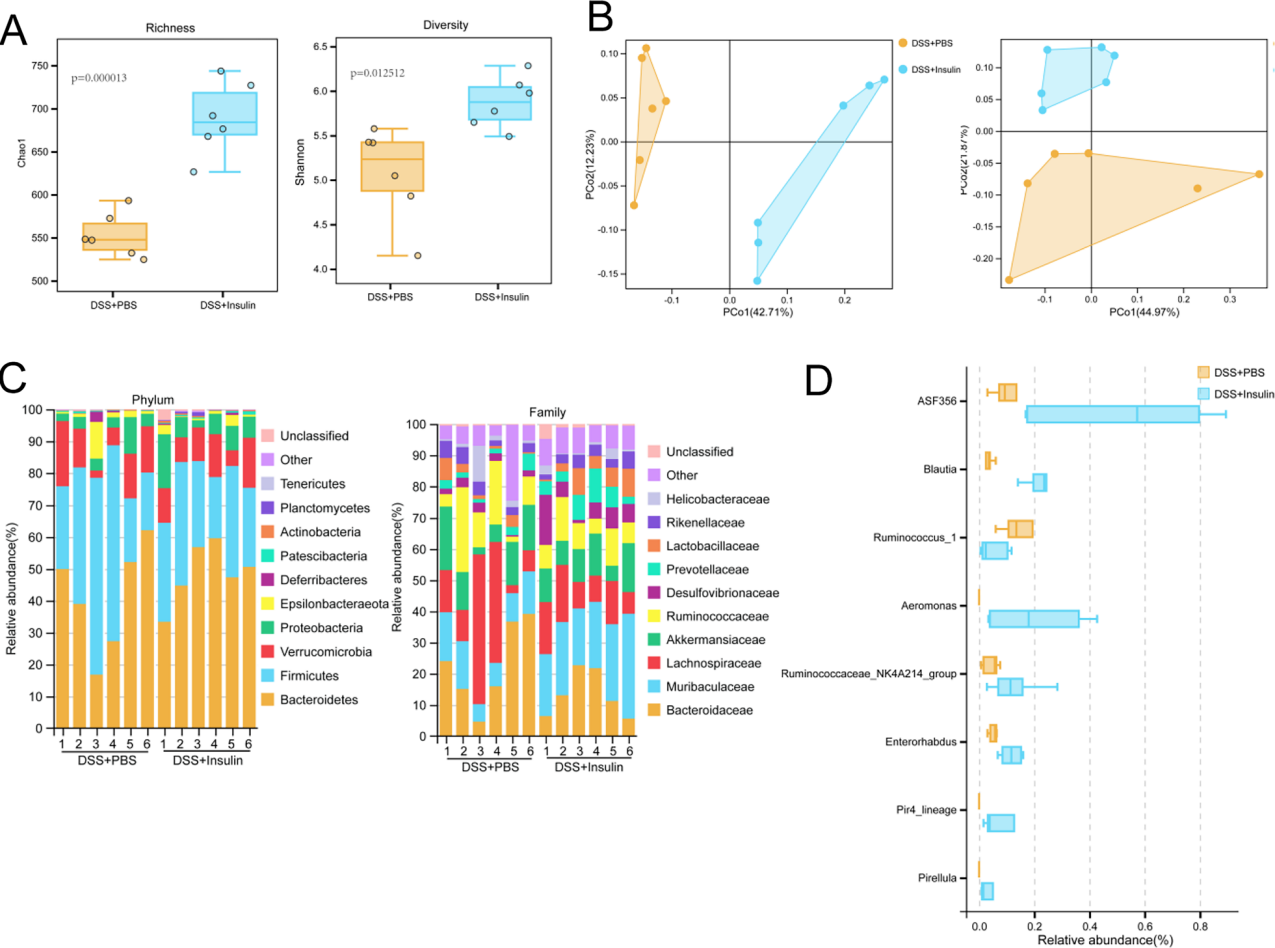
Insulin modulates the intestinal microbiota of DSS-treated mice. WT mice were treated with DSS for 5 days, followed by 3 days of normal drinking water, and the mice were treated with PBS (n = 6) or insulin (n = 6) intraperitoneally on day 3, day 5 and day 7. **A** Microbial richness and diversity in the PBS- and insulin-treated mice based on the Chao1 index and the Shannon index. **B** The impact of insulin on the β-diversity of microbial communities was analyzed by unweighted UniFrac metrics and weighted UniFrac metrics. **C** Relative abundances of OTUs at the phylum and family levels in the gut microbiota of all the groups. **D** Relative abundances of OTUs of the genera *Ruminococcaceae*, *Lachnospiraceae*, *Pirellulaceae*, *Ruminococcus_1*, *ASF356*, *Blautia*, *Aeromonas*, *Ruminococcaceae_NK4A214_group*, *Enterorhabdus*, *Pir4_lineage* and *Pirellula*

To determine whether treatment with insulin alters the microbial community in the gut, 16S rRNA sequencing analysis of fecal samples collected from DSS-treated mice that were treated with insulin was conducted. Analysis of α-diversity using the Chao1 and Shannon indices showed that insulin significantly increased species richness (*P* = 0.000013) and diversity (*P* = 0.012512) (Fig. 3A). The impact of insulin on the β-diversity of microbial communities was analyzed by unweighted UniFrac metrics and weighted UniFrac metrics, and the results showed that insulin-treated mice exhibited significant alterations, as reflected by distinct clustering patterns on principal coordinate analysis (PCoA) plots (Fig. 3B). Then, we characterized the relative abundances of different bacteria after insulin treatment. Insulin treatment profoundly influenced multiple bacterial families. At the phylum level, the relative abundance of Bacteroidetes was increased, while that of Firmicutes was decreased (Fig. 3C). At the family level, insulin treatment increased the relative abundance of *Muribaculaceae*, whereas those of *Bacteroidaceae*, *Lachnospiraceae*, *Ruminococcaceae* and *Lactobacillaceae* were reduced in insulin-treated mice (Fig. 3C). To identify species that characterized the insulin group vs. the PBS group, we performed indicator analysis. The results showed that most of the altered bacteria belonged to *Ruminococcaceae*, *Lachnospiraceae* and *Pirellulaceae* and that the relative abundance of *Ruminococcus_1* was significantly decreased, whereas the relative abundances of *ASF356*, *Blautia*, *Aeromonas*, *Ruminococcaceae_NK4A214_group*, *Enterorhabdus*, *Pir4_lineage* and *Pirellula* were significantly increased in the insulin group compared with the PBS group (Fig. 3D).

**Fig. 4:**
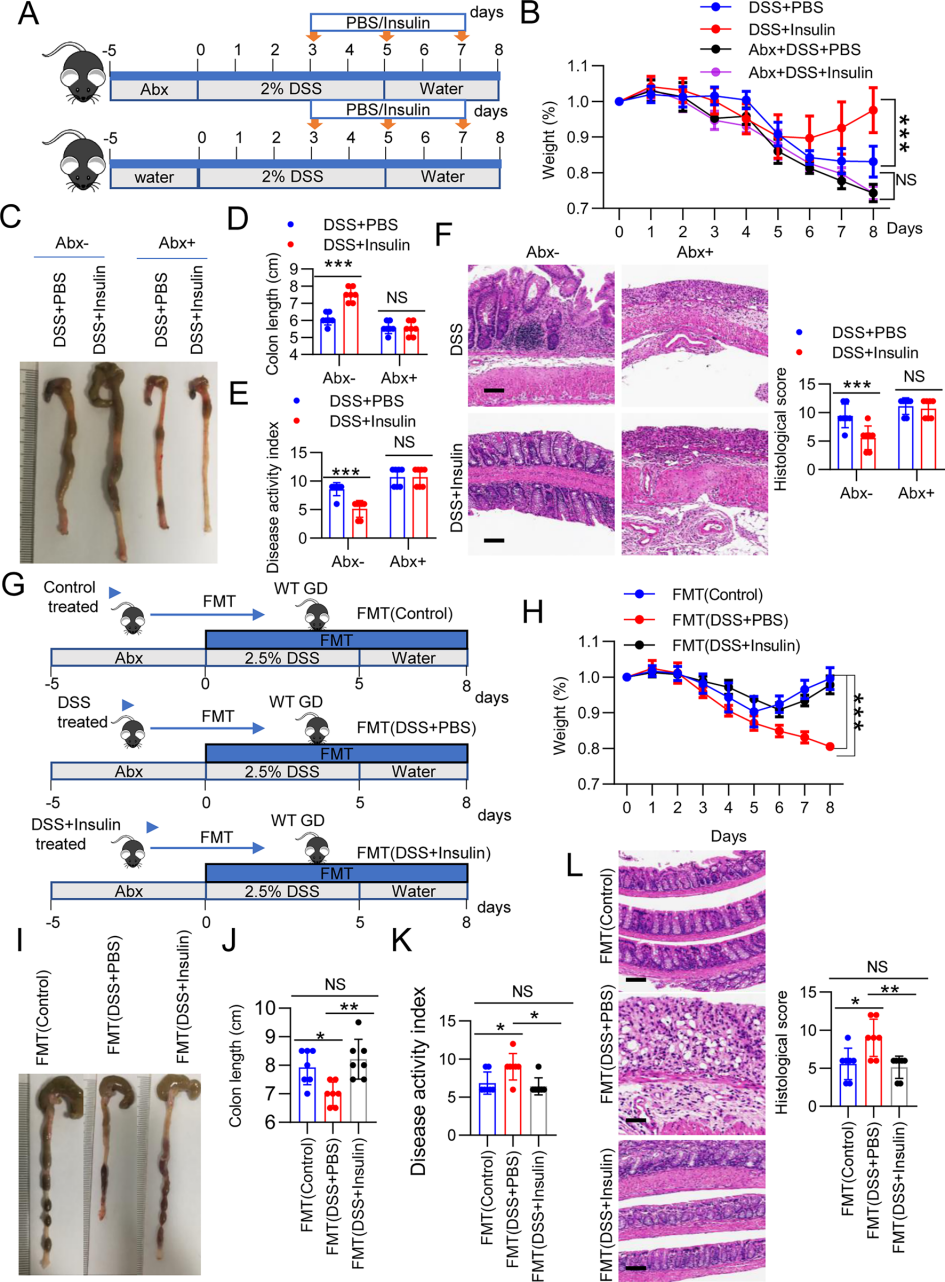
The insulin-mediated reduction in colitis pathogenesis is dependent on the gut microbiota. **A**–**F** Abx intervention significantly decreased the abundance of bacteria. Mice were treated with or without Abx for 5 days. Then, the water was changed to 2.5% DSS, and the mice were treated with insulin (n = 7 per group). **B** Body weight changes were monitored daily after DSS administration. **C** Colons were harvested from mice treated with PBS and insulin. **D** Colon length and **E** DAI scores in all the groups. **F** Representative images of H&E staining of colon sections from different treatment groups. Scale bar, 100 μm. **G**–**L** WT mice were treated with Abx for 5 days, administered 2.5% DSS and underwent FMT of feces originating from the normal group, insulin-treated DSS group and PBS-treated DSS group (n = 7 per group). **H** Body weight changes were monitored daily after DSS administration. **I** Colons were harvested from mice treated with PBS and insulin. **J** Colon length and **K** DAI scores in all the groups. **L** Representative images of H&E staining of colon sections from different treatment groups. Scale bar, 100 μm

Given the significant differences in gut microbial communities after insulin treatment, we wanted to determine whether the effects of insulin on colitis could be attributed to the gut microbiota. First, we pretreated mice with antibiotics (Abx) and then treated them with DSS (Fig. 4A), and the depletion of the microbiota was confirmed by qPCR (Additional file 1: Fig. S3). Consistent with the phenomena observed in the animal models described in Fig. 1, insulin treatment promoted body weight regain and reduced the DAI score and colon histopathology (Fig. 4B–F). However, mice treated with Abx showed aggravated colitis disease parameters, as shown by assessment of weight loss, colon length, and colon histopathology (Fig. 4B–F). More importantly, among mice treated with Abx, no significant differences in body weight, colon length, DAI score or colon morphology were observed between the insulin-treated and PBS-treated mice (Fig. 4B–F). To further investigate the effect of the gut microbiota on the insulin-alleviated colitis phenotypes in the DSS model, mice were pretreated with Abx, and then the intestinal microbiota collected from normal mice (control), PBS-treated mice with DSS-induced colitis or insulin-treated mice with DSS-induced colitis was transplanted into mice by oral gavage. Simultaneously, acute colitis was induced in the recipient mice by DSS treatment (Fig. 4G). After DSS withdrawal, the FMT (control) (mice that received the control microbiota) and FMT (DSS + insulin) (mice transplanted with the microbiota from the insulin-treated DSS group) groups exhibited improvements in colitis, as shown by rapid body weight recovery, longer colon lengths and reduced DAI scores (Fig. 4H–K), compared to the FMT (DSS + PBS) (mice transplanted with the microbiota from PBS-treated DSS group) group. Accordingly, the FMT (control) and FMT (DSS + insulin) groups also showed significantly reduced leukocyte infiltration and histological inflammation in the colon (Fig. 4L and Additional file 1: Fig. S4). Collectively, these experiments demonstrate that the microbiota of insulin-treated mice was able to protect against DSS-induced colonic inflammation.

### Restructuring of bile acid metabolism by insulin

**Fig. 5:**
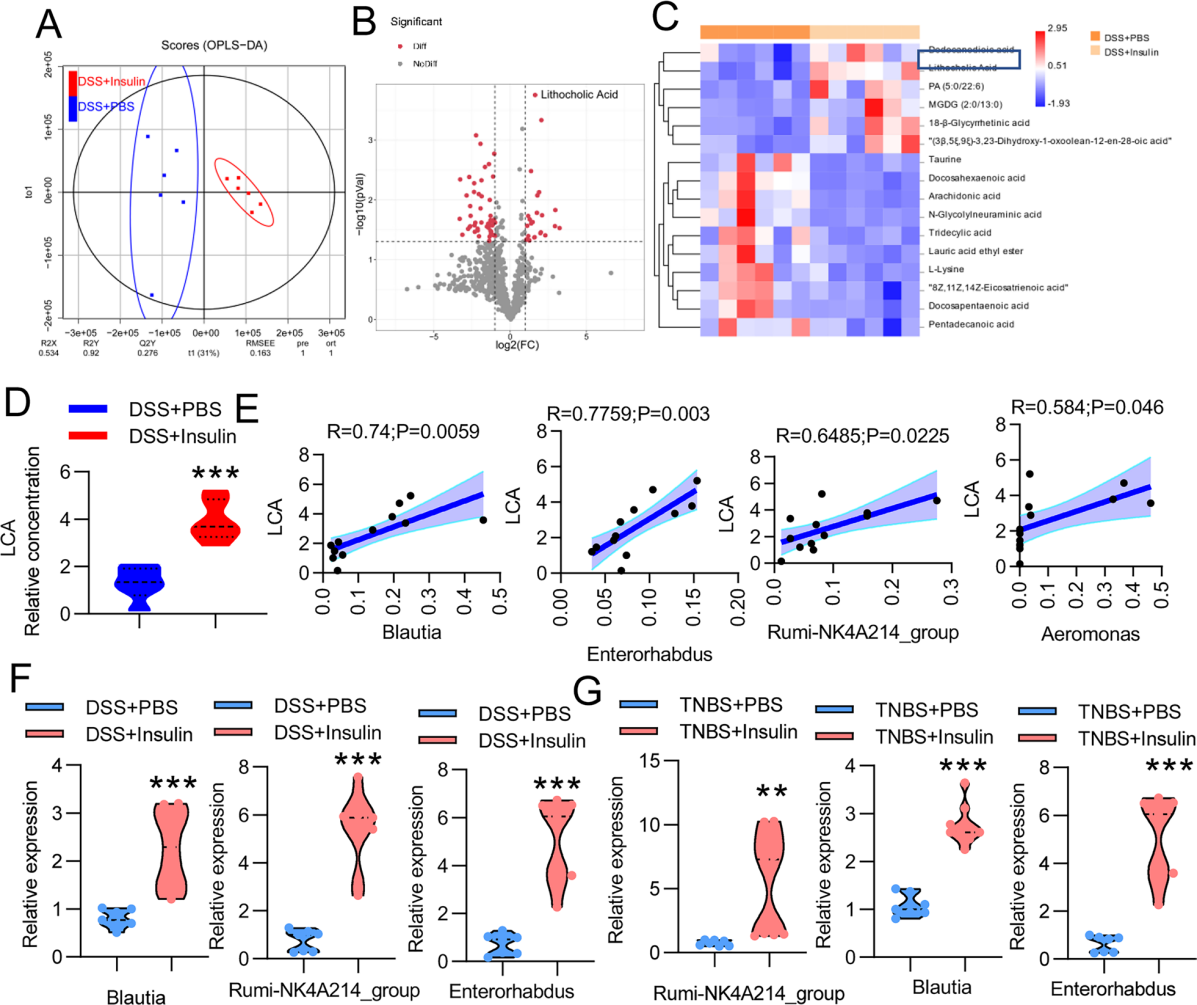
SBA metabolism was increased by insulin. Mice were given 5 days of DSS treatment and then were treated with PBS (n = 6) or insulin (n = 6) on day 3, day 5 and day 7. **A** Orthogonal projections to latent structures-discriminant analysis (PLS-DA) score plot for discriminating the intestinal digesta metabolome from the PBS- and insulin-treated groups. **B** Volcano plot showing the differential abundance of intestinal digesta metabolites between PBS- and insulin-treated mice. **C** Heatmaps of the differential metabolites whose levels were altered by insulin treatment compared with PBS treatment. **D** Comparison of the relative abundance of LCA in the indicated groups. **E** Spearman’s correlation analysis of LCA levels with *Blautia*, *Enterorhabdus*, *Ruminococcaceae_NK4A214_group* and *Aeromonas* abundance. **F**, **G** The relative abundances of *Blautia*, *Enterorhabdus* and *Ruminococcaceae_NK4A214_group* in the indicated groups were determined by real-time PCR. The data represent the mean ± SD. **P < 0.01; ***P < 0.001

Gut-derived metabolites play a key role between the gut microbiota and host. To gain a more comprehensive understanding of metabolites that may be involved in the ameliorative effect of insulin on colitis, we performed untargeted metabolomic profiling using UPLC–MS analysis. Using partial least squares discriminant analysis, we observed distinct clustering of metabolites between PBS- and insulin-treated mice with DSS-induced colitis (Fig. 5A). Further analyses identified 23 upregulated and 27 downregulated metabolites in insulin-treated DSS-induced mice compared with PBS-treated mice with DSS-induced colitis (Fig. 5B). Interestingly, the most strikingly increased metabolite was LCA (Fig. 5B–D). We also performed targeted metabolomic profiling of the bile acid levels in the gut contents of mice treated with DSS alone, and the LCA levels were significantly decreased in the DSS group compared with the control group (Additional file 1: Fig. S5A). These results show that the elevated LCA level observed following insulin intervention may contribute to the control of colitis.

To further investigate the effects of microbes on LCA levels, Spearman’s correlation analysis was performed. As shown in Fig. 5E, Spearman’s correlation analysis between LCA and differential genus-level bacteria revealed that the bacteria *Blautia*, *Enterorhadus*, *Rumi-NK4A214_group* and *Aeromonas* were significantly positively correlated with LCA. *Blautia*, *Enterorhadus* and *Rumi-NK4A214_group* have been reported to be capable of metabolizing bile acids and are closely related to bile acid 7α-dehydroxylating species (resulting in SBAs) [34, 43, 44]. These data suggest that *Blautia*, *Enterorhadus* and *Rumi-NK4A214_group* are involved in insulin-regulated LCA production. To confirm this phenomenon, we measured the abundances of *Blautia*, *Enterorhadus* and *Rumi-NK4A214_group* in the mice with DSS-induced colitis, and the abundances of *Blautia*, *Enterorhadus* and *Rumi-NK4A214_group* were dramatically decreased in the DSS group (Additional file 1: Fig. S5B). In addition, we detected the abundances of *Blautia*, *Enterorhadus* and *Rumi-NK4A214_group* in the context of insulin treatment, and they all were dramatically increased in the insulin-treated DSS and insulin-treated TNBS groups (Fig. 5F, G). Furthermore, we found that the abundance of LCA in the FMT (control) and FMT (DSS + insulin) groups was significantly increased compared with that in the FMT (DSS + PBS) group (Additional file 1: Fig. S5C). We also detected the abundances of *Blautia*, *Enterorhadus* and *Rumi-NK4A214_group* in feces from the three groups and found that the abundances of *Blautia*, *Enterorhadus* and *Rumi-NK4A214_group* were dramatically increased in the FMT (control) and FMT (DSS + insulin) groups (Additional file 1: Fig. S5D). Thus, LCA is significantly associated with the compositional changes in the microbiota in insulin-treated colitis mice.

### Insulin ameliorates intestinal inflammation by increasing the level of LCA

**Fig. 6:**
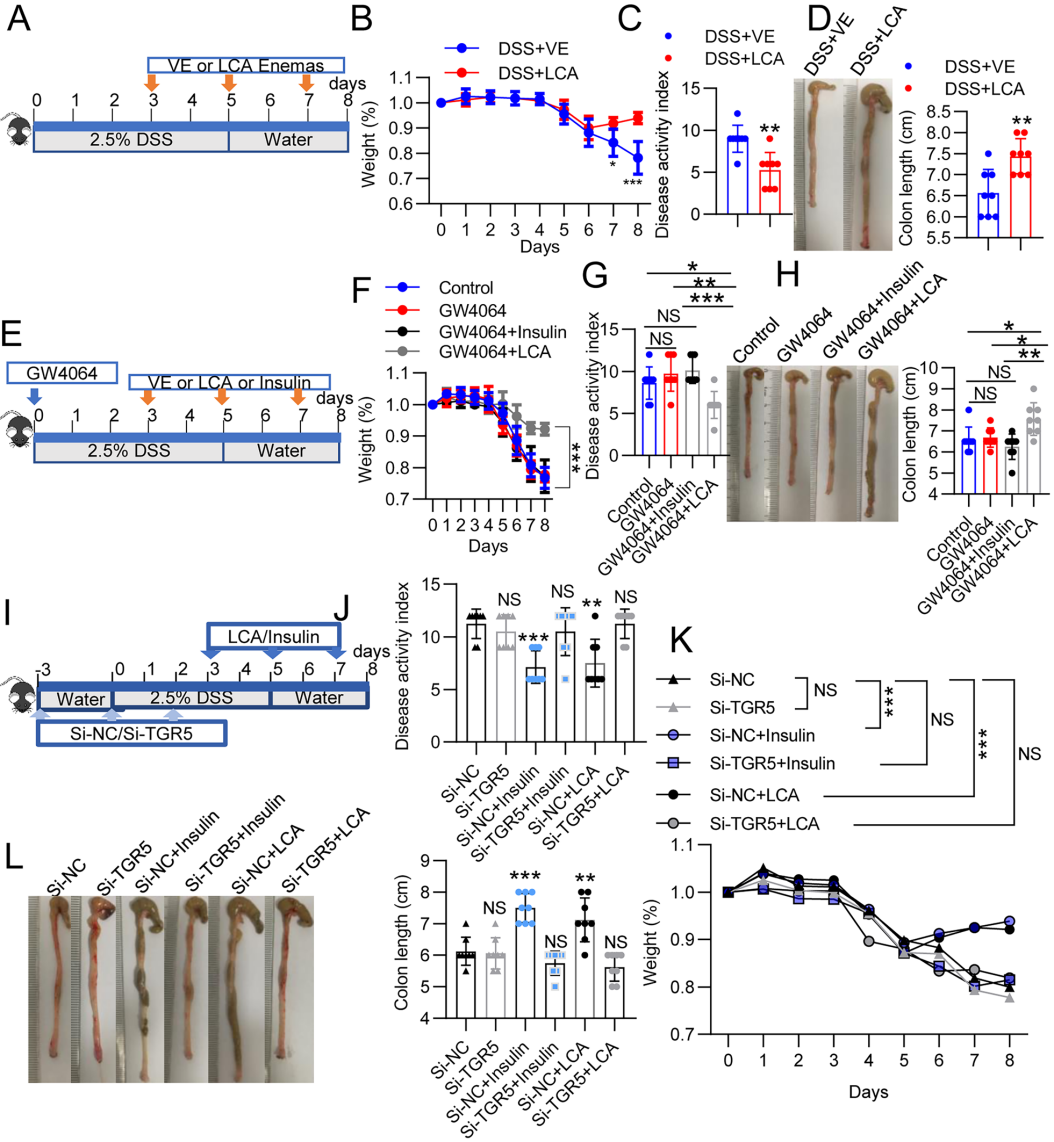
Insulin alleviated colitis through the LCA-Tgr5 pathway. **A**–**D** Mice were given water supplemented with 2.5% DSS and were treated with 100 μL of bile acid suspension (5 mg LCA) or vehicle control (VE) via the rectum on days 3, 5, and 7. **B** Changes in body weight were monitored daily after DSS administration. **C** DAI scores and **D** colon length in all the groups. **E**–**H** GW4064 was orally administered at a dose of 10 mg/kg twice with a 12-h interval between doses. Then, 2.5% DSS was given to the mice, and insulin and LCA were administered. **F** Body weight, **G** DAI scores and **H** colon length of the mice in the control, GW4064, GW4064 + insulin, and GW4064 + LCA groups. **I**–**L** Mice were administered methylated siRNA targeting Tgr5 by intraperitoneal injection (1 OD/mouse) on days -3, 0 and 2 of the experiment. Three days after the first administration of siRNAs, 2.5% DSS was administered to the mice, and then, the mice were treated with insulin and LCA. **J** Body weight, **K** DAI scores and **L** colon length in all the groups. The data represent the mean ± SD. NS, not significant; *P < 0.05; **P < 0.01; ***P < 0.001

The LCA level is significantly increased in the gut contents of insulin-treated mice with DSS-induced colitis compared to those of PBS-treated mice with DSS-induced colitis, which has also been demonstrated to alleviate colitis [32]. Therefore, we treated DSS-treated mice with LCA (5 mg/mouse) via oral administration (Fig. 6A). LCA reproducibly reduced colitis disease, as reflected by weight loss, disease activity, colon length, and gross colon morphology results, compared with the vehicle control (Fig. 6B–D and Additional file 1: Fig. S6A). To investigate whether depletion of LCA in insulin-treated mice impacts colitis, we treated mice with DSS-induced colitis with GW4064, a farnesoid X receptor (FXR) agonist, via oral gavage to suppress hepatic bile acid synthesis (Fig. 6E). GW4064 treatment did not influence colitis, as indicated by the body weight, colon length, DAI scores and histological damage in the GW4064 group compared with the control group (Fig. 6F–H and Additional file 1: Fig. S6B). Further treatment with LCA in DSS-induced colitis attenuated colitis, as indicated by reduced body weight loss, DAI scores, and histological scores and increased colon length (Fig. 6F–H and Additional file 1: Fig. S6B). However, GW4064-treated mice that received insulin showed significantly increased DAI scores (Fig. 6G), reduced weight (Fig. 6F), reduced colon length (Fig. 6H), and gut damage marked by infiltration of mononuclear cells and neutrophils into the lamina propria (Additional file 1: Fig. S6B). Therefore, we concluded that insulin alleviated colitis by mediating LCA production.

FXR and TGR5 are bile acid receptors, and LCA is the most potent bile acid ligand for TGR5 activation [45]. Thus, we hypothesized that insulin alleviates colitis in the DSS model through the LCA-TGR5 axis. To validate this hypothesis, we pretreated all the mice with si-NC or si-TGR5 and then treated them with DSS, insulin and LCA on days 3, 5 and 7 after beginning DSS treatment (Fig. 6I). The results showed that the decreased DAI scores (Fig. 6J) and histological damage (Additional file 1: Fig. S6C) as well as the increased body weight (Fig. 6K) and colon length (Fig. 6L) induced by insulin and LCA in the si-NC-treated mice were significantly eliminated in the si-TGR5-treated mice (Additional file 1: Fig. S7). These experiments highlight the contribution of the LCA-TGR5 axis to insulin-mediated protection against DSS-induced colitis.

### Insulin regulates M1 macrophage polarization in the colon

**Fig. 7:**
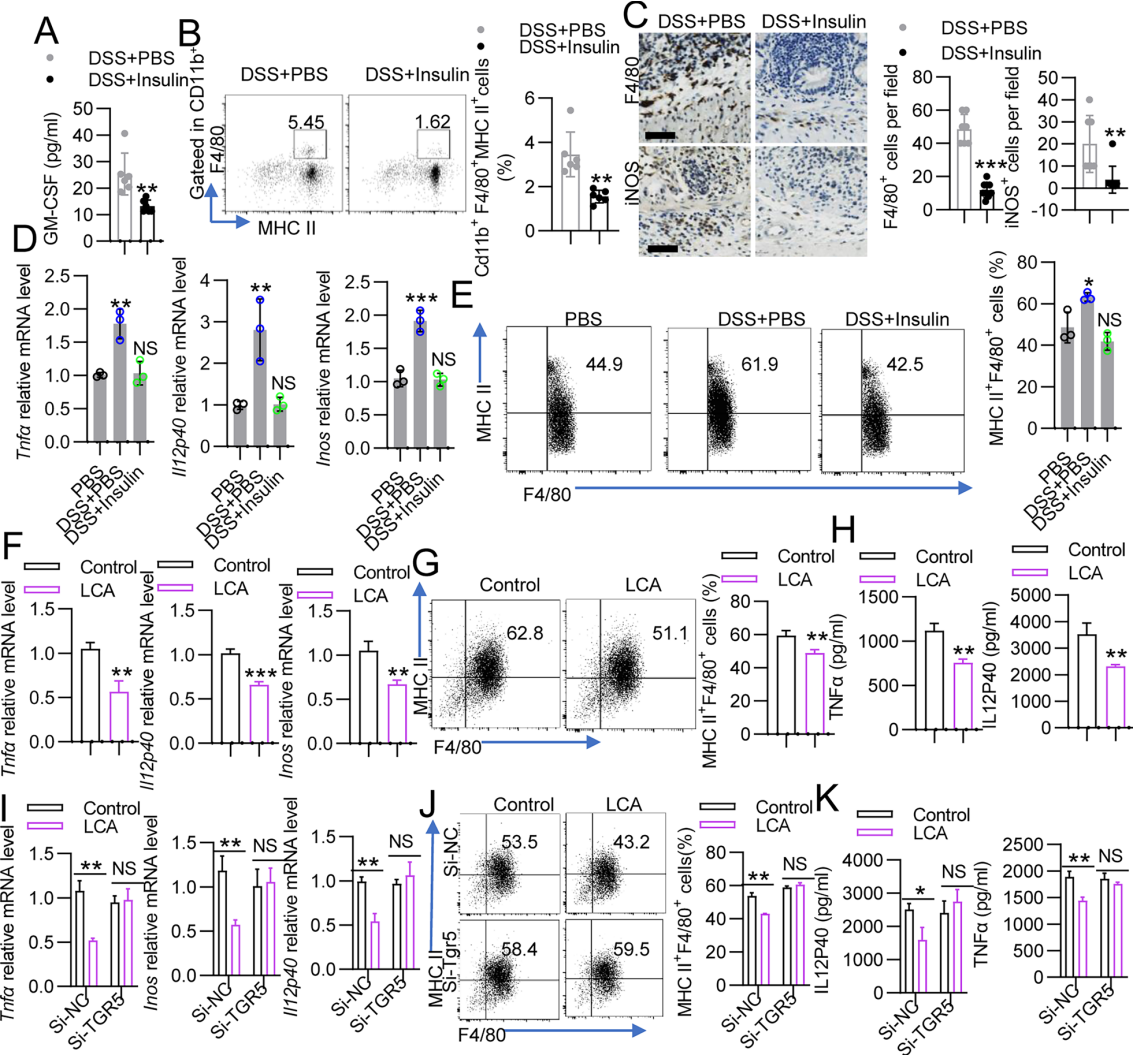
LCA alters macrophage polarization through TGR5. **A**–**C** Mice were treated with DSS for 5 days, followed by 3 days of normal drinking water, and the mice were treated with PBS or insulin intraperitoneally on day 3, day 5 and day 7. **A** Cytokine concentrations in the serum were measured by Luminex. **B** Flow cytometry analysis of the numbers of CD11b^+^Ly6C^+^Ly6G^−^ cells in the colon. **C** Representative images of F4/80 and iNOS immunohistochemical staining in sections of colon tissue. Scale bar = 50 μm. The supernatants of the gut contents from the PBS (normal mice), DSS + PBS and DSS + insulin groups were added to treat BMDMs, which were then treated with LPS plus IFNγ. **D** The levels of proinflammatory factors (*Tnfα*, *Il12p40* and *inos*) and **E** the numbers of F4/80^+^MHC II^+^ cells were measured by qPCR and FACS, respectively. BMDMs were treated with LCA (50 μM) for 12 h and then were stimulated with IFNγ plus LPS for 4 h or 24 h. **F** The levels of proinflammatory factors (*Tnfα*, *Il12p40* and *inos*), **G** numbers of F4/80^+^MHC II^+^ cells and **H** the cell supernatant concentrations of IL-10 and TGFβ1 were measured. BMDMs from WT mice were transfected with si-NC and si-Tgr5 for 12 h, and the cells were then stimulated with LPS/IFNγ or LCA. **I** The levels of proinflammatory factors (*Tnfα*, *Il12p40* and *inos*), **J** numbers of F4/80^+^MHC II^+^ cells and **K** the cell supernatant concentrations of IL-10 and TGFβ1 were measured. The data represent the mean ± SD. NS, not significant; *P < 0.05; **P < 0.01; ***P < 0.001

Next, we aimed to determine the mechanisms underlying the protective effects of insulin and LCA treatment. To discover whether insulin alleviates colitis by regulating the inflammatory process, serum was collected from insulin-treated mice with DSS-induced colitis and evaluated with Luminex assays. Notably, the concentrations of C-X-C motif chemokine 1 (CXCL1) and TNFα were not significantly altered (Additional file 1: Fig. S8). However, the concentrations of IL-6, CXCL2 and monocyte chemoattractant protein 1 (MCP-1), which are principal produced by macrophages [46], were decreased in the insulin-treated group compared with the PBS-treated group (Additional file 1: Fig. S8). Interestingly, the production of granulocyte–macrophage colony-stimulating factor (GM-CSF), which induces macrophage differentiation and survival, was significantly suppressed by insulin treatment (Fig. 7A). Thus, we investigated whether macrophage accumulation in the colon is influenced. Indeed, M1 macrophages (CD11b^+^F4/80^+^MHC II^+^) were markedly decreased in insulin-treated colons compared to PBS-treated colons (Fig. 7B and Additional file 1: Fig. S9A). Treg and Th17 cells have also been linked to secondary bile acid metabolism [47]. Thus, we also detected Tregs and Th17 cells. However, the Th1, Treg and Th17 cells in the colon were not changed by insulin (Additional file 1: Fig. S9B-C). Furthermore, the protein expression of F4/80 and inducible nitric oxide synthase (iNOS), which are markers of macrophages and M1 macrophages, respectively, was examined in colon tissues from mice treated with or without insulin by IHC staining. The colon tissues from mice treated with insulin were found to express significantly lower levels of F4/80 and iNOS than those from mice treated with PBS (Fig. 7C). Hence, insulin was shown to alleviate colitis by inhibiting M1 macrophage polarization in the colon.

To further confirm that the inhibition of M1 macrophage polarization in the insulin-treated group was due to alterations in the microbes colonizing the gut and relevant bile acid levels, we first examined the F4/80^+^ and iNOS^+^ cells in the colons of the model animals described in Fig. 1G. The IHC staining results showed that F4/80^+^ and iNOS^+^ cells were significantly decreased in the FMT (control) and FMT (DSS + insulin) groups compared to the FMT (DSS + PBS) group (Additional file 1: Fig. S10A). Next, we examined whether the supernatants of the gut contents affect macrophage polarization by comparing the PBS (normal mice), DSS + insulin and DSS + PBS groups. Gut material was prepared following a strict procedure involving dilution and homogenization and then used to treat BMDMs that were stimulated in vitro with interferon γ (IFNγ) plus lipopolysaccharide (LPS). We found that the levels of proinflammatory factors (*Tnfα*,* Il12p40* and *inos*) and the numbers of M1 macrophages (F4/80^+^MHC II^+^), as determined by FACS, were not significantly different between the PBS and DSS + insulin groups (Fig. 7D, E). However, the mRNA expression of the M1 macrophage signature molecules *Tnfα*, *Il12p40* and *inos* was significantly reduced in M1 macrophages treated with supernatants from the DSS + insulin group compared with those treated with supernatants from the DSS + PBS group (Fig. 7D). Furthermore, F4/80^+^MHC II^+^ cells were also significantly reduced (Fig. 7E). Collectively, these data suggest that insulin treatment impairs M1 macrophage polarization in colon tissues during colitis.

### The LCA-TGR5 axis reprograms macrophages in the colon

Then, we asked whether insulin influences M1 macrophage polarization through LCA. LCA treatment decreased the number of F4/80^+^ and iNOS^+^ cells in the colons of insulin-treated mice with DSS-induced colitis (Additional file 1: Fig. S10B). To determine whether LCA directly regulates macrophage polarization, we analyzed proinflammatory factors and F4/80^+^MHC II^+^ cells among murine BMDMs stimulated in vitro with IFNγ plus LPS. The mRNA expression of M1 markers (*Tnfα*, *Il12p40* and *inos*) was deregulated in LCA-treated M1 macrophages (Fig. 7F), and the percentages of F4/80^+^MHC II^+^ cells were decreased (Fig. 7G). These findings correlated with the decreased secretion of TNFα and IL12P40 observed by LCA-treated macrophages, as determined by ELISA (Fig. 7H). Consistently, the negative effect of insulin on the reduced M1 macrophage infiltration in the colons of insulin-treated mice was abrogated by GW4604 treatment (Additional file 1: Fig. S10C). However, GW4604 did not influence the inhibitory effect of LCA on M1 macrophage infiltration into the colon (Additional file 1: Fig. S10C). Next, we confirmed that LCA reduced M1 macrophage through the bile acid receptor TGR5. si-TGR5 was transfected into BMDMs to knockdown TGR5 expression. Knocking down TGR5 expression abrogated the effect of LCA on M1 macrophages, as shown by the increased mRNA expression of *Tnfα*, *Il12p40* and *inos*, increased numbers of F4/80^+^MHC II^+^ cells and enhanced secretion of TNFα and IL12P40 (Fig. 7I–K). Furthermore, knocking down TGR5 expression abrogated the effects of insulin and LCA on macrophages during colitis, as shown by the increased numbers of F4/80^+^ and iNOS^+^ cells in the colon (Additional file 1: Fig. S11A-B). Together, these data suggest that LCA reprograms M1 macrophages through TGR5.

## Discussion

Our hypothesis in the present study was that insulin regulates colitis by modifying the gut microbiota. This hypothesis was based on three observations reported in a previous study: (1) metformin is associated with a low risk of IBD in diabetes mellitus patients [28], (2) rectal insulin instillation reduces colon inflammation through the insulin receptor [12], and (3) the gut microbiota can influence the effect of hypoglycemic agents [27, 48]. Overall, our study demonstrates that insulin alleviates the development of colitis in murine IBD models and that this effect is mediated by altering the gut microbiota. Furthermore, by using a multiomic approach to examine the compositions of the microbiota and metabolome, we identified that LCA metabolism was improved after insulin treatment, which was modulated by some bacterial strains.

The goal of IBD therapy is to induce and maintain remission. Medical therapy for IBD includes drugs such as immunomodulators and targeted biologic therapies [49]. However, some patients usually experience fewer potential side effects or declare themselves to have an aggressive disease. More drugs for IBD remain to be found. Previous research showed that insulin inhibits intestinal inflammation by enema [12]. To further explore the mechanism by which insulin alleviates colitis, we used insulin to treat murine colitis models, including DSS- and TNBS-induced colitis models. In our study, we tested whether insulin exerts an effect on intestinal inflammation via intraperitoneal injection because insulin is usually injected subcutaneously in the clinic. In our study, a low dose of insulin reduced the blood glucose concentration to 3.0–4.0 mmol/L, and it did not cause death of the mice. Moreover, we found that insulin treatment significantly reduced colitis. The importance of glycolysis in IBD has been demonstrated in a previous study [50], where inhibition of glycolysis using 2-deoxyglucose decreased the inflammatory response. However, we did not observe a similar effect in the mice that received insulin treatment; thus, the anti-inflammatory effect is probably attributable to insulin itself. However, how to prevent and manage hypoglycemia in IBD patients, especially in those without diabetes, maybe a major challenge associated with the clinical use of in insulin. Future studies are warranted to confirm this observation.

We hypothesized that the effect of insulin in colitis involved alterations in the gut microbiota. We tested our hypothesis that the effects of insulin on murine colitis models involved alterations in the gut microbiota. We validated this hypothesis with Abx and FMT experiments, and the protective effect of insulin was abrogated by Abx treatment. Furthermore, we transplanted fecal contents from PBS- and insulin-treated mice with DSS-induced colitis. This experimental approach allowed for direct examination of the effects of the cecal content, presumably bacteria, in the absence of genetic differences between PBS- and insulin-treated mice with DSS-induced colitis [51]. In support of our hypothesis, we showed that mice receiving the microbiota of insulin-treated donors had significantly alleviated colitis compared to those transplanted with the microbiota of PBS-treated control donors. In our study, 16S rRNA sequencing analysis revealed that the gut microbiota structure was significantly different between the PBS- and insulin-treated DSS groups. Importantly, we found that insulin increased the relative abundance of *Muribaculaceae*, whereas those of *Bacteroidaceae*, *Lachnospiraceae*, *Ruminococcaceae* and *Lactobacillaceae* were reduced. In addition to the fecal microbiota, other factors may also participate in the ameliorative effect of insulin on colitis.

Next, we performed untargeted metabolomic analysis of the fecal contents of the two groups. Notably, our data showed that LCA was the molecule with the greatest difference in expression between the insulin group and the PBS group, and SBAs have been reported to promote the maintenance of mucosal integrity by increasing intestinal epithelial cell migration [52] and inhibiting epithelial apoptosis [53, 54] and epithelial regeneration by activating intestinal stem cells [55], thus alleviating acute and chronic enteropathy [25, 32]. This finding suggests that SBAs may play a role in the insulin-induced reduction in intestinal inflammation. We confirmed the ability of LCA to mitigate the effects of colitis, and insulin alleviated colitis in part through LCA. The Clostridiales order and Ruminococcaceae family are known to possess the requisite 7a-dehydroxylating capability to generate SBAs [23]. In fact, *Clostridium hiranonis sp*. have been shown to generate SBAs much more efficiently [56]. Moreover, our correlation analysis suggested that the relative abundances of *Blautia*, *Enterorhadus* and *Rumi-NK4A214_group* were positively correlated with LCA production, which has been reported to be related to bile acid 7α-dehydroxylating species, which can induce SBA production [34, 43, 44]. On the other hand, *Blautia*, *Enterorhadus* and *Rumi-NK4A214_group* were enriched by insulin treatment. These results showed that the altered gut microbiota and associated metabolites caused by insulin intervention might play a central role in colitis.

Finally, we found that insulin alleviated colonic inflammation, as exemplified by the declines in the levels of inflammatory cytokines such as GM-CSF and IL-6 in the plasma of mice with DSS-induced colitis, and GM-CSF is a factor that induces macrophage differentiation and survival [57]. LCA has been demonstrated to suppress proinflammatory cytokine production in macrophages through activation of the receptor TGR5 [58]. Thus, we explored the molecular mechanism by which insulin ameliorates colitis through modulation of the gut microbiota and associated metabolites. LCA alleviated colitis and even inhibited M1 macrophage polarization in vivo and in vitro. These results provide clues for the development of a potential new strategy for colitis treatment using insulin and LCA as therapeutic agents. The ameliorative effect of LCA was further examined in TGR5 knockdown mice, and the results revealed that insulin and LCA ameliorated colitis development through TGR5. Insulin may, therefore, exert anti-inflammatory functions by modulating the gut microbiota and SBA production.

In conclusion, insulin could be a therapeutic agent with the potential to be developed into a functional anti-inflammatory agent to ameliorate colitis (as shown in the Graphical Abstract).

## Supplementary Information

**Additional file 1.** **Materials and methods**: **Table S1**: Sequences of siRNAs and primers used in this study. **Table S2**: Antibody list. **Figure S1**: Insulin ameliorated acute colitis in a murine model. **Figure S2**: Abx intervention significantly decreased the abundance of bacteria. **Figure S3**: WT mice were treated with Abx for 5 days, and the mice were administered 2.5% DSS and underwent FMT. **Figure S4**: The level of LCA was improved by fecal transplants of the microbiota of insulin-treated mice. **Figure S5**: Colon mucosal histology in different groups. **Figure S6**: Mice were administered methylated siRNA targeting Tgr5 by intraperitoneal injection. **Figure S7**: Cytokine concentrations in the serum were measured by Luminex. **Figure S8**: Gating strategy for flow cytometric analysis of immune cell populations. **Figure S9**: Insulin inhibited M1 macrophage polarization through the LCA-Tgr5 pathway. **Figure S10**: LCA inhibited M1 macrophage polarization through TGR5.

## Data Availability

All data are available in the main text or the Supplementary Materials. The sequencing raw data are available upon request.

## Abbreviations

IBD: Inflammatory bowel disease
UC: Ulcerative colitis
CD: Crohn’s disease
FMT: Fecal microbiota transplantation
SBAs: Secondary bile acids
LCA: Lithocholic acid
M1: Classically activated macrophage
TGR5: G-protein-coupled receptor 5
PBS: Phosphate-buffered saline
DSS: Dextran sulfate sodium
DAI: Disease activity index
IL: Interleukin
TNF: Tumor necrosis factor
ZO-1: Zonula occludens 1
TNBS: 2,4,6-Trinitrobenzenesulfonic acid solution
LGR5: Leucine-rich repeat-containing receptor 5
PCoA: Principal coordinate analysis
Abx: Antibiotics
FXR: Farnesoid X receptor
CXCL1: C-X-C motif chemokine 1
MCP1: Monocyte chemoattractant protein 1
GM-CSF: Granulocyte-macrophage colony-stimulating factor
iNOS: Inducible nitric oxide synthase
IFNγ: Interferon γ
LPS: Lipopolysaccharide
