# Supplementary material for: Insulin alleviates murine colitis through microbiome alterations and bile acid metabolism
Source: J Transl Med. 2023 Jul 25;21:498. doi: 10.1186/s12967-023-04214-3 (PMC10369930; doi:10.1186/s12967-023-04214-3)
Supplement: Supplementary file 1 — Additional file 1. Materials and methods: Table S1: Sequences of siRNAs and primers used in this study. Table S2: Antibody list. Figure S1: Insulin ameliorated acute colitis in a murine model. Figure S2: Abx intervention significantly decreased the abundance of bacteria. Figure S3: WT mice were treated with Abx for 5 days, and the mice were administered 2.5% DSS and underwent FMT. Figure S4: The level of LCA was improved by fecal transplants of the microbiota of insulin-treated mice. Figure S5: Colon mucosal histology in different groups. Figure S6: Mice were administered methylated siRNA targeting Tgr5 by intraperitoneal injection. Figure S7: Cytokine concentrations in the serum were measured by Luminex. Figure S8: Gating strategy for flow cytometric analysis of immune cell populations. Figure S9: Insulin inhibited M1 macrophage polarization through the LCA-Tgr5 pathway. Figure S10: LCA inhibited M1 macrophage polarization through TGR5. [file 12967_2023_4214_MOESM1_ESM.docx]

**Supplementary Material**

**Insulin alleviates murine colitis through microbiome alterations and bile acid metabolism**

Shuying He^1^, Jiating Li^1^, Zirong Yao^1^, Zixian Gao^1^, Yonghong Jiang^1^, Xueqing Chen^1#^, Liang Peng^1#^

1 Department of Gastroenterology, First Affiliated Hospital of Guangzhou Medical University, Guangzhou Medical University, Guangzhou, China

**Correspondence to:**

Professor Liang Peng, Department of Gastroenterology, First Affiliated Hospital of Guangzhou Medical University

Mailing address: First Affiliated Hospital of Guangzhou Medical University, No. 151, Yanjiang West Road, Yuexiu District, Guangzhou, Guangdong, 510120, P.R. China

Tel: +862083062090

Fax: +862083062090

E-mail: wsfirefly@126.com

Professor Xueqing Chen, Department of Gastroenterology, First Affiliated Hospital of Guangzhou Medical University

Mailing address: First Affiliated Hospital of Guangzhou Medical University, No. 151, Yanjiang West Road, Yuexiu District, Guangzhou, Guangdong, 510120, P.R. China

Tel: +862083062090

Fax: +862083062090

E-mail: chenxq@vip.163.com

Supplementary Table 1. Sequences of siRNAs and primers used in this study

| Sequences of siRNAs used in this study | | |
| --- | --- | --- |
| Gene | Sense | Antisense |
| Control siRNA | UUCUCCGAACGUGGUCACGU | ACGUGACACGUUCGGAGAA |
| TGR5 | GCUUCUUCCUAAGCCUAUTT | AGUAGGCUUAGGAAGAAGCTT |

| Sequences of primers used for real-time PCR | | |
| --- | --- | --- |
| Gene | Primer (Forward) | Primer (Reverse) |
| Occludin | GCTGTGATGTGTGTGAGCTG | GACGGTCTACCTGGAGGAAC |
| Reg IIIγ | TTCCTGTCCTCCATGATCAAAA | CATCCACCTCTGTTGGGTTCA |
| Reg IIIβ | TGGGAATGGAGTAACAATG | GGCAACTTCACCTCACAT |
| Zo-1 | AGGACACCAAAGCATGTGAG | GGCATTCCTGCTGGTTACA |
| Lgr5 | AGCGTCTTCACCTCCTACCT | ATCTAGGCGCAGGGATTGAAG |
| S100a9 | CAAAGGCTGTGGGAAGTAATTAAG | TTGAGTAAGCCATTCCCTTTAGAC |
| Il1β | GACGGCACACCCACCCT | AAACCGTTTTTCCATCTTCTTCTTT |
| Il12p40 | ACAGCACCAGCTTCTTCATCAG | TCTTCAAAGGCTTCATCTGCAA |
| Il6 | GTAGCTATGGTACTCCAGAAGAC | ACGATGATGCACTTGCAGAA |
| Inos | CCAGCACTTCACCCATCAGTT | AAGGCGCAGTTTATGTTGTCTGT |
| Tnfα | GCCACCACGCTCTTCTGTCT | GGTCTGGGCCATAGAACTGATG |
| Gapdh | TCCCACTCTTCCACCTTCGATGC | GGGTCTGGGATGGAAATTGTGAGG |
| Hif1α | CCTGCACTGAATCAAGAGGTTGC | CCATCAGAAGGACTTGCTGGCT |
| Slc2a4 | GGTGTGGTCAATACGGTCTTCAC | AGCAGAGCCACGGTCATCAAGA |
| Hk2 | CCCTGTGAAGATGTTGCCCACT | CCTTCGCTTGCCATTACGCACG |
| Ldha | ACGCAGACAAGGAGCAGTGGAA | ATGCTCTCAGCCAAGTCTGCCA |
| Tpi1 | GGCAACTGGAAGATGAACGGGA | CTGGCAAAGTCGATGTAAGCGG |
| Gpi1 | CCATCAAGGTGGACGGCAAAGA | CCGTGATGGATTTGCCAGTGTAC |
| Eno2 | TGGCAAGGATGCCACTAACGTG | AACTCAGAGGCAGCCACATCCA |
| Eno3 | CTGCTCCTGAAGGTCAACCAGA | GTCAGCGATGAAAGTGTCTTCGG |
| Pfk1 | CCATCAGCAACAATGTGCCTGG | TGAGGCTGACTGCTTGATGCGA |
| Pgm1 | AGCCAATGACCCAGATGCTGAC | TCCAGGAAGTGAAGAGCCACCA |
| Pgk1 | GATGCTTTCCGAGCCTCACTGT | ACCAGCCTTCTGTGGCAGATTC |
| Hprt | CTGGTGAAAAGGACCTCTCGAAG | CCAGTTTCACTAATGACACAAACG |
| Rumi-NK4A214_group | AACTCATAAACTGCATTTGAAACTGTACT | AGCGTCAGTTGCTGTCCAGTAGAC |
| Aeromonas | GAAGGCCAAGTCGGCCGCCAG | ATCTTGGCATCGCCCGGGTTTTC |
| Blautia | TCTGATGTGAAAGGCTGGGGCTTA | GGCTTAGCCACCCGACACCTA |
| Enterorhabdus | ATGGCTGTCGTCAGCTCGT | CCTACTTCTTTTGCAACCCACTC |
| 16S (V2) | AGYGGCGIACGGGTGAGTAA | CYIACTGCTGCCTCCCGTAG |
| 16S (V6) | AGGATTAGATACCCTGGTA | CRRCACGAGCTGACGAC |
| Frimicute | GGAGYATGTGGTTTAATTCGAAGCA | AGCTGACGACAACCATGCAC |
| Bacteroides | CTGAACCAGCCAAGTAGCG | CCGCAAACTTTCACAACTGACTTA |
| Beta-Proteobacteria | AACGCGAAAAACCTTACCTACC | TGCCCTTTCGTAGCAACTAGTG |
| Epsilon-  Proteobacteria | TAGGCTTGACATTGATAGAATC | CTTACGAAGGCAGTCTCCTTA |
| Delta-Gamma  Proteobacteria | GCTAACGCATTAAGTRYCCCG | GCCATGCRGCACCTGTCT |

Table S2 Antibody list

| Antibody | Source | Identifier |
| --- | --- | --- |
| eFluor506-conjugated anti-mouse CD3 | eBioscience | 69-0032-82 |
| FITC-conjugated anti-mouse CD4 | eBioscience | 11-0041-82 |
| BV421-conjugated anti-mouse IL17A | BioLegend | 506926 |
| Percp-Cy5.5-conjugated anti-mouse interferon gamma (IFNγ) | eBioscience | 45-7311-82 |
| APC-conjugated anti-mouse forkhead box protein 3 (FOXP3) | eBioscience | 17-5773-82 |
| PE-conjugated anti-mouse MHC II | eBioscience | 12-5322-81 |
| FITC-conjugated anti-mouse F4/80 | eBioscience | 11-4801-82 |
| PE-Cy7-conjugated anti-mouse CD11b | eBioscience | 25-0112-82 |


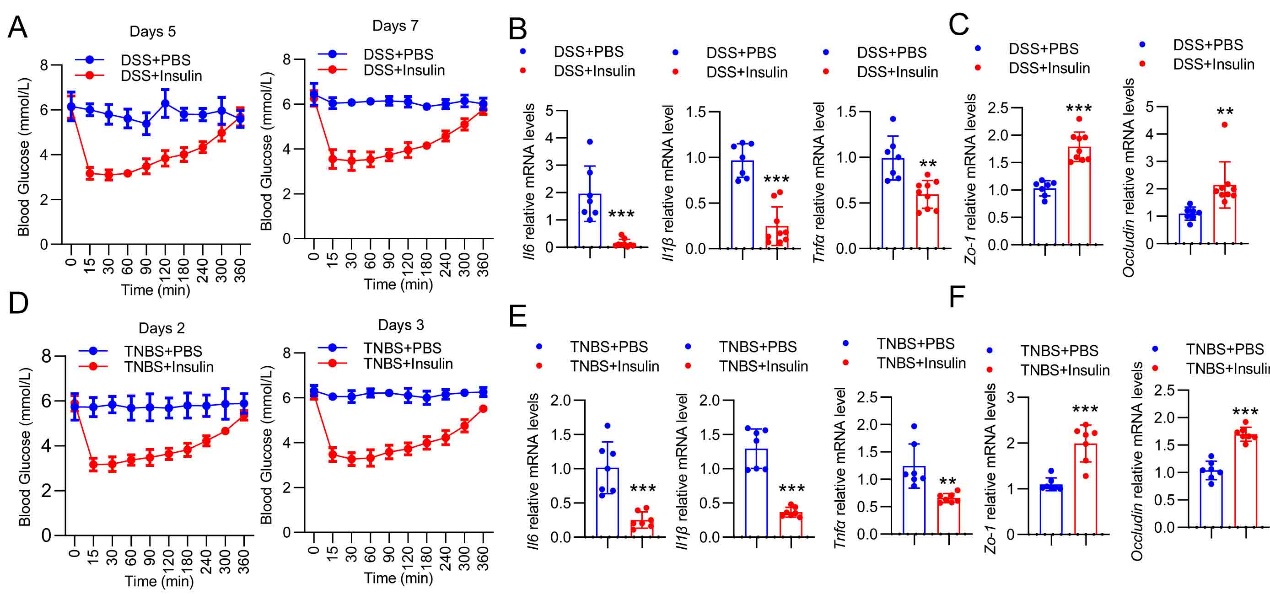


**Supplementary Figure 1.** Insulin ameliorated acute colitis in a murine model. **(A-C)** WT mice were treated with DSS for 5 days, followed by 3 days of normal drinking water, and the mice were treated with PBS (n=7) or insulin (n=9) intraperitoneally on days 3, 5 and 7. **(A)** Blood glucose levels were determined on days 5 and 7. **(B-C)** mRNA expression levels of the indicated genes in the colonic mucosa of PBS- and insulin-treated mice. **(D-F)** Mice were presensitized with topical TNBS, further sensitized via the rectotransfer of TNBS, and treated with PBS or insulin on day 1, day 2 and day 3 (n=7 in each group). **(D)** Blood glucose levels were determined on days 2 and 3. **(E-F)** mRNA expression levels of the indicated genes in the colonic mucosa of PBS- and insulin-treated mice treated with TNBS. The data represent the mean ± SD. **P<0.01; ***P<0.001.


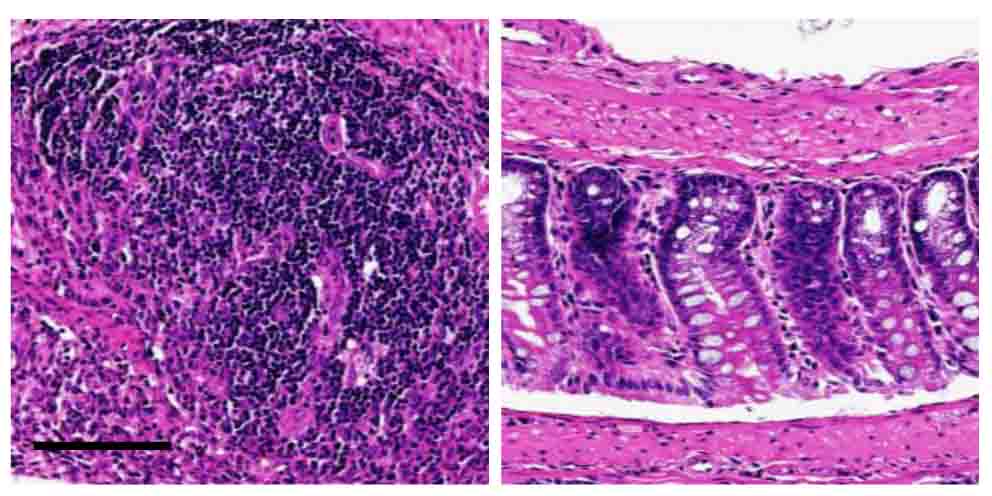


**Supplementary Figure 2.** WT mice were treated with DSS for 5 days, and then given normal drinking water for 3 days, and the mice were treated with PBS (n=7) or insulin (n=9) intraperitoneally on day 3, day 5 and day 7. Paraffin-embedded sections of PBS- and insulin-treated mouse colons were analyzed by H&E. Scale bar, 50 μm.


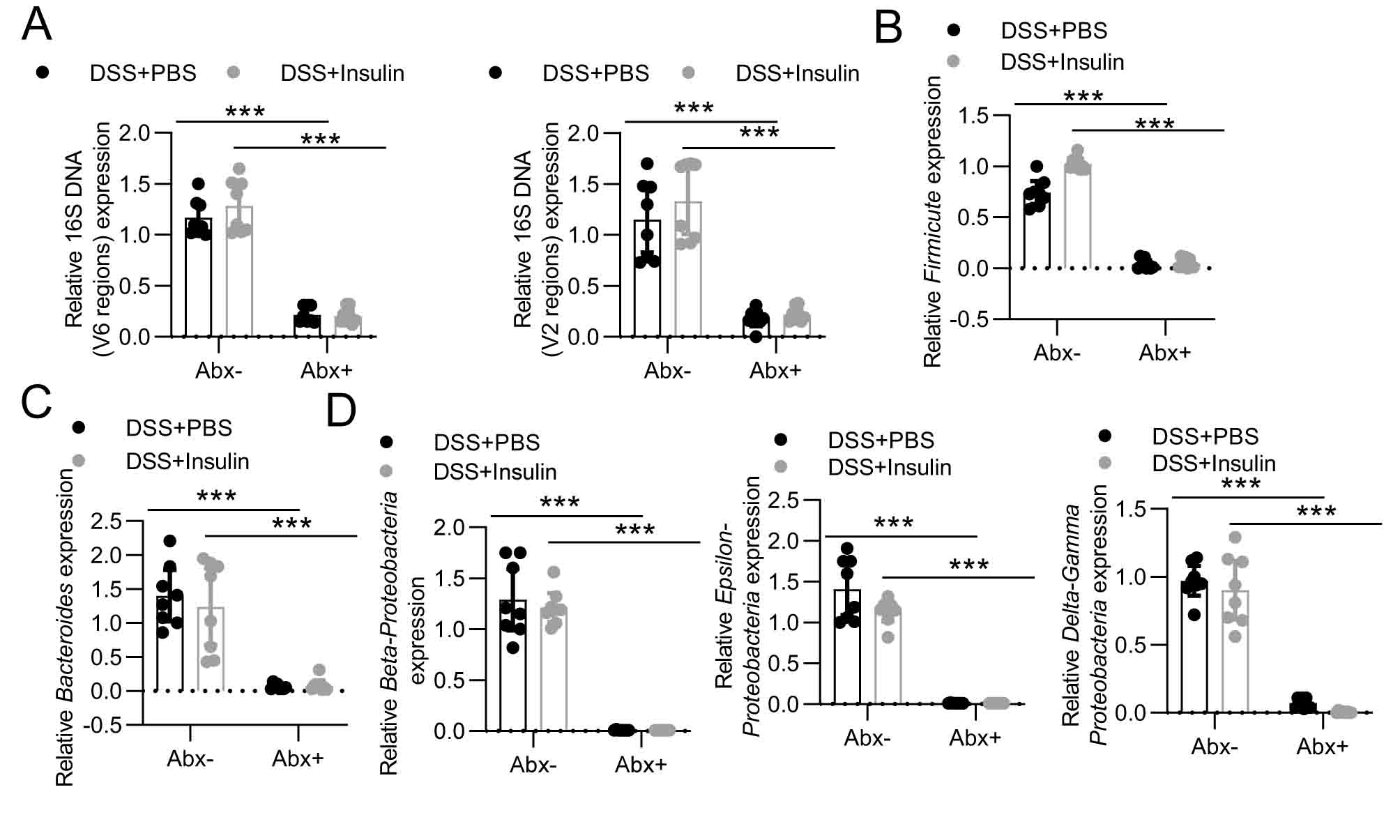


**Supplementary Figure 3.** Abx intervention significantly decreased the abundance of bacteria. Mice were treated with or without Abx for 5 days. Then, the water was changed to 2.5% DSS, and the mice were treated with insulin (n=7 per group). **(A)** Genetic DNA was extracted from the microbiota of the feces, and qPCR was used to analyze the level of 16S rDNA (V2 and V6 regions) relative to the level of the mouse pIgR genomic region. The abundance of **(B)** *Firmicutes*, **(C)** *Bacteroidetes*, **(D)** *Betaproteobacteria*, *Epsilonproteobacteria* and *Deltaproteobacteria-* and *Gammaproteobacteria* was assessed based on 16S by qPCR. The data are expressed as the mean ± SD. ***P<0.001.


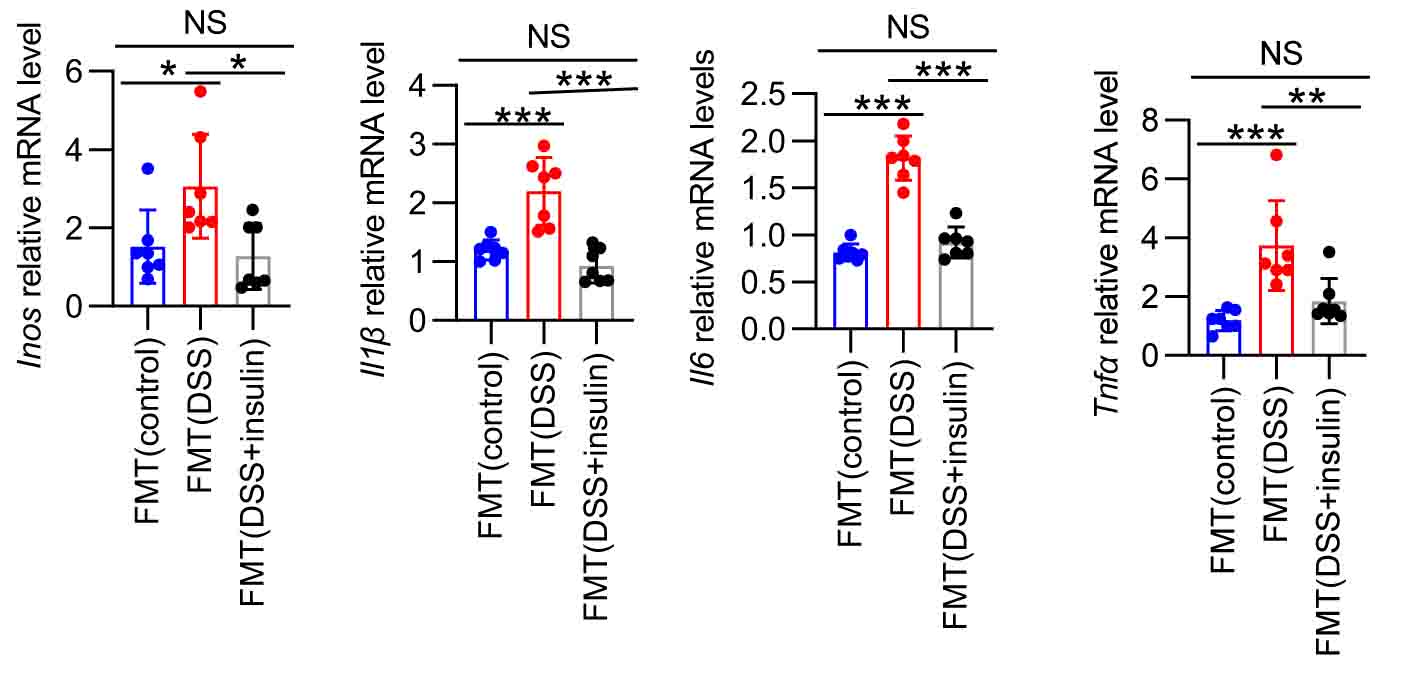


**Supplementary Figure 4.** WT mice were treated with Abx for 5 days, and the mice were administered 2.5% DSS and underwent FMT of feces originating from the normal group, insulin-treated DSS group and PBS-treated DSS group (n=7 per group). mRNA expression levels of the indicated genes in the colonic mucosa of all the groups. The data represent the mean ± SD. NS, not signiﬁcant; *P<0.05; **P<0.01; ***P<0.001.


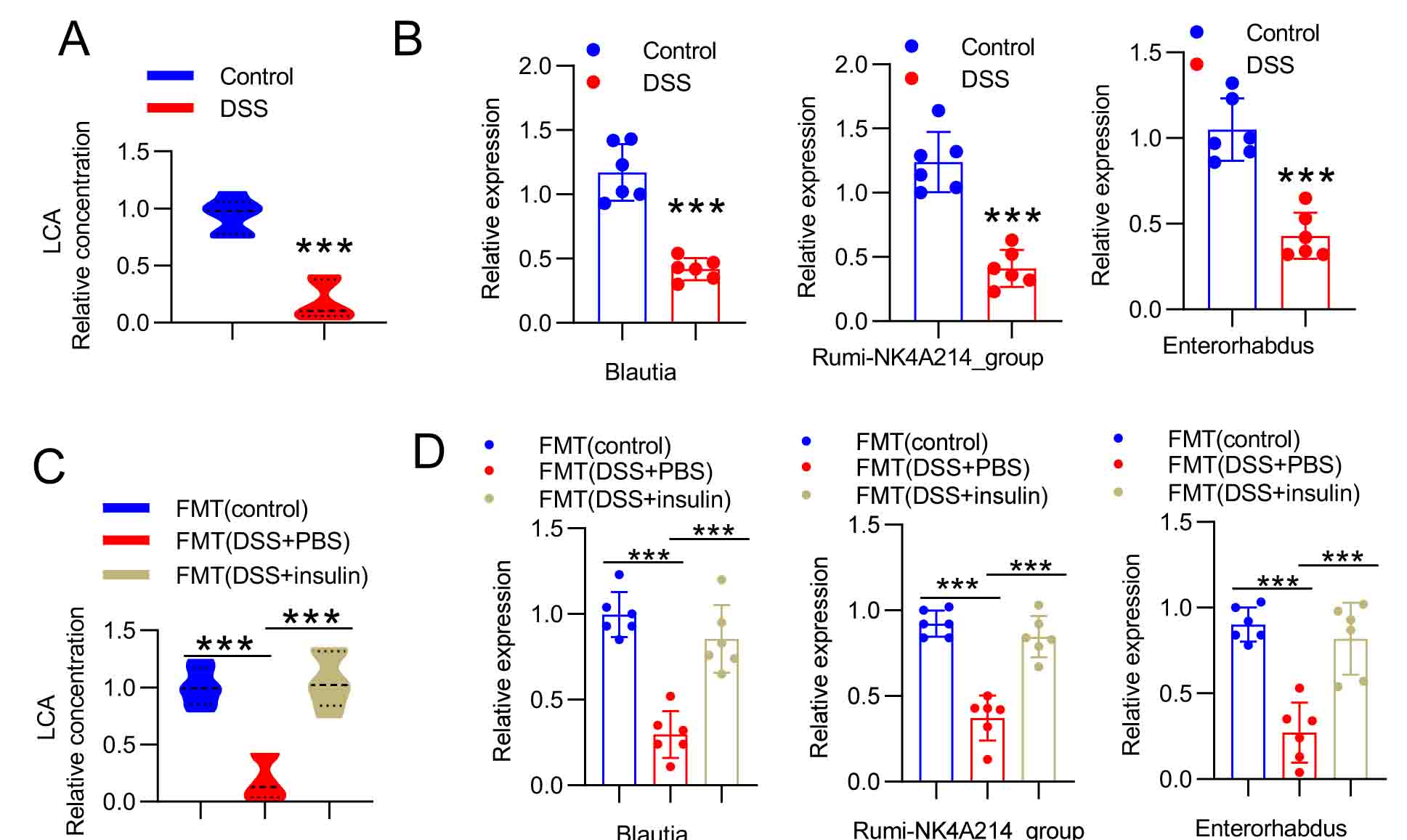


**Supplementary Figure 5.** The level of LCA was improved by fecal transplants of the microbiota of insulin-treated mice. **(A)** LCA levels in DSS-treated mice were measured by targeted metabolomics. **(B)** The relative abundance of *Blautia*, *Enterorhabdus* and *Ruminococcaceae_NK4A214_group* in the DSS group was determined by real-time PCR. **(C)** LCA levels were measured by targeted metabolomics in the indicated groups. **(D)** The relative abundances of *Blautia*, *Enterorhabdus* and *Ruminococcaceae_NK4A214_group* were determined by real-time PCR in the indicated groups. The data represent the mean ± SD. ***P<0.001.


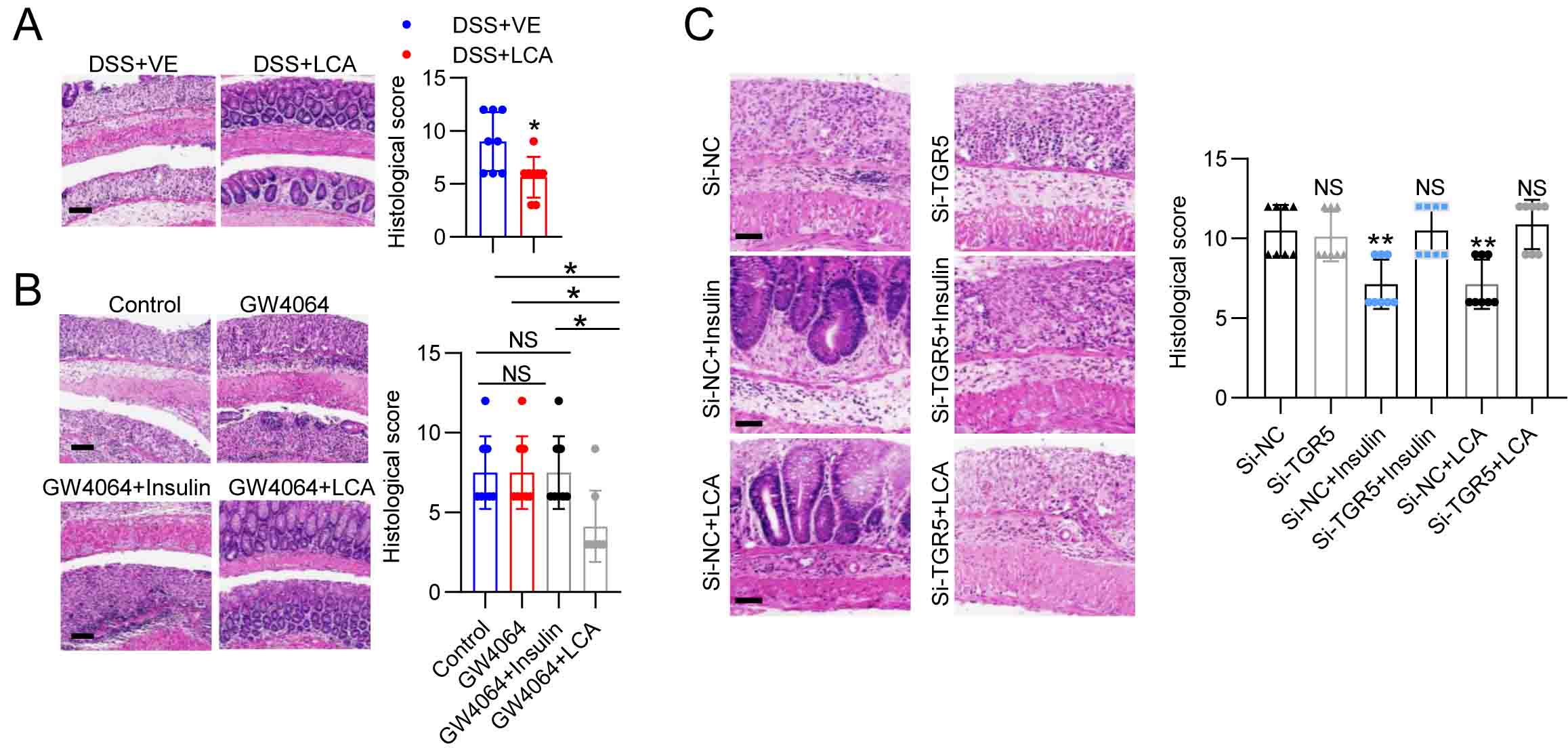


**Supplementary Figure 6.** Colon mucosal histology in different groups. **(A)** Mice were given water supplemented with 2.5% DSS and treated with a 100 μL suspension of bile acid (5 mg of LCA) or vehicle control (VE) via the rectum on days 3, 5, and 7. Representative images of H&E staining of colon sections from different treatment groups. Scale bar, 100 μm. **(B)** GW4064 was orally administered at a dose of 10 mg/kg twice with a 12 h interval between doses. Then, 2.5% DSS was administered to the mice, and the mice were treated with insulin and LCA. Representative images of H&E staining of colon sections from different treatment groups. Scale bar, 100 μm. **(C)** Mice were administered methylated siRNA targeting Tgr5 by intraperitoneal injection (1 OD/ mouse) on days -3, 0 and 2 of the experiment. Three days after the first administration of siRNAs, 2.5% DSS was administered, and the mice were treated with insulin and LCA. Representative images of H&E staining of colon sections from different treatment groups. Scale bar, 100 μm. The data represent the mean ± SD. NS, not signiﬁcant; *P<0.05; **P<0.01.


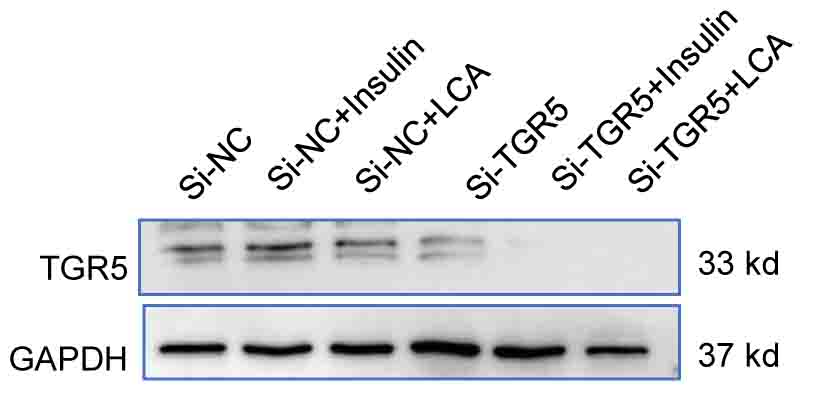


**Supplementary Figure 7.** Mice were administered methylated siRNA targeting Tgr5 by intraperitoneal injection. Three days after the first administration of siRNAs, 2.5% DSS was administered, and the mice were treated with insulin and LCA. Tgr5 expression in the colon was measured by western blotting.


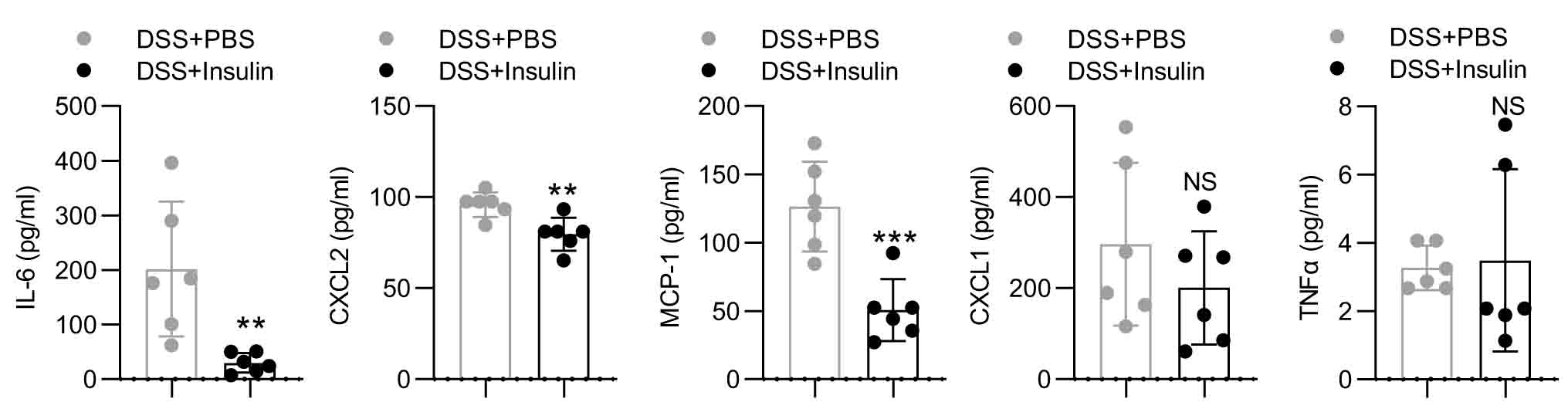


**Supplementary Figure 8.** Mice were treated with DSS for 5 days, followed by 3 days of normal drinking water, and the mice were treated with PBS or insulin intraperitoneally on day 3, day 5 and day 7. Cytokine concentrations in the serum were measured by Luminex. The data represent the mean ± SD. NS, not signiﬁcant; **P<0.01; ***P<0.001.


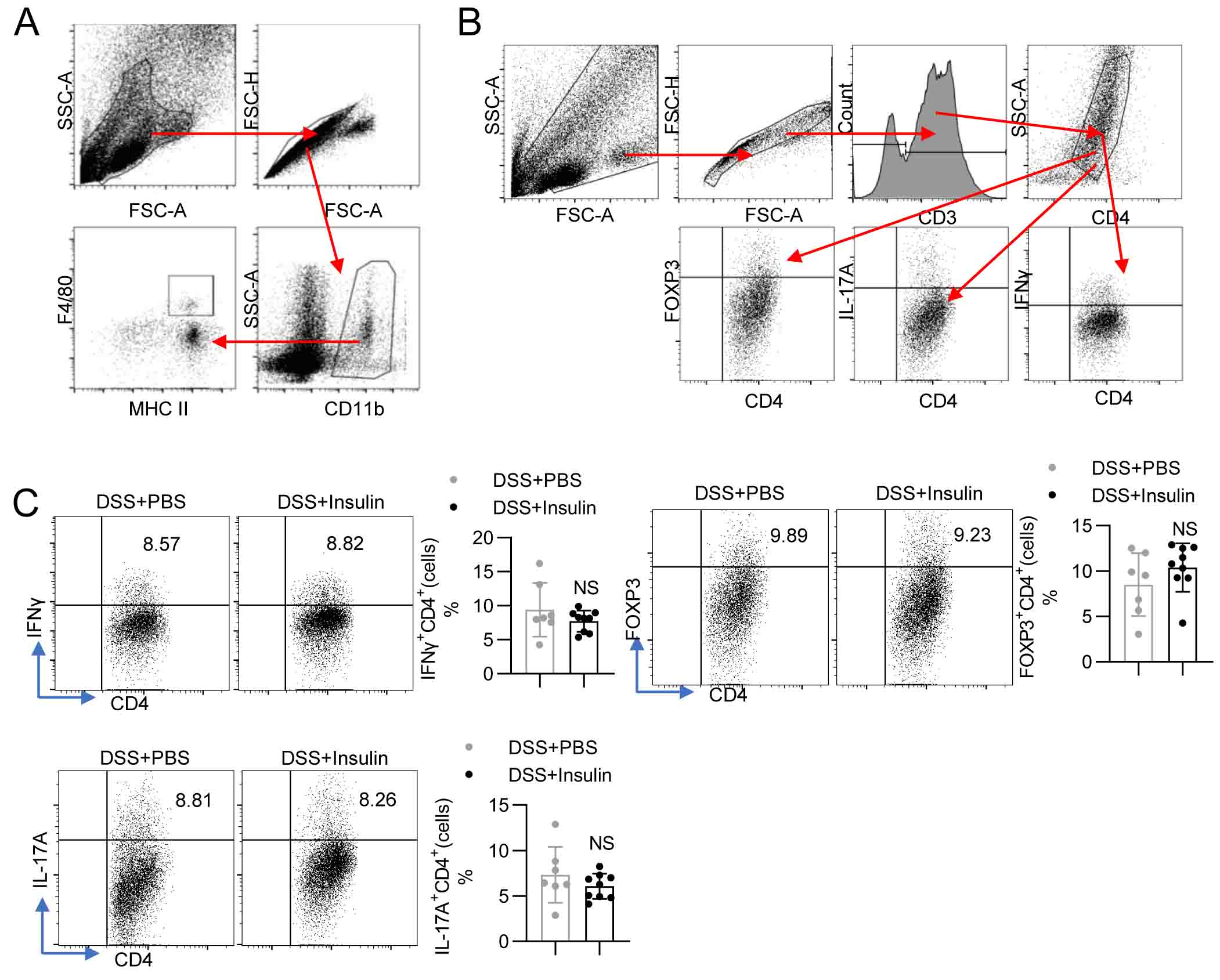


**Supplementary Figure 9.** Gating strategy for flow cytometric analysis of immune cell populations. **(A)** Doublets were removed, and CD11b^+^ cells were then further assessed as F4/80^+^MHC II^+^ cells. **(B)** Doublets were removed, and CD4+ T cells were then further assessed as IL-17^+^CD4^+^, IFNγ^+^CD4^+^ and FOXP3^+^CD4^+^ cells. **(C)** Flow cytometry analysis of IL-17+CD4+, FOXP3+CD4+ and IFN-γ+CD4+ cells in the colon by intracellular staining. The data represent the mean ± SD. NS, not signiﬁcant.


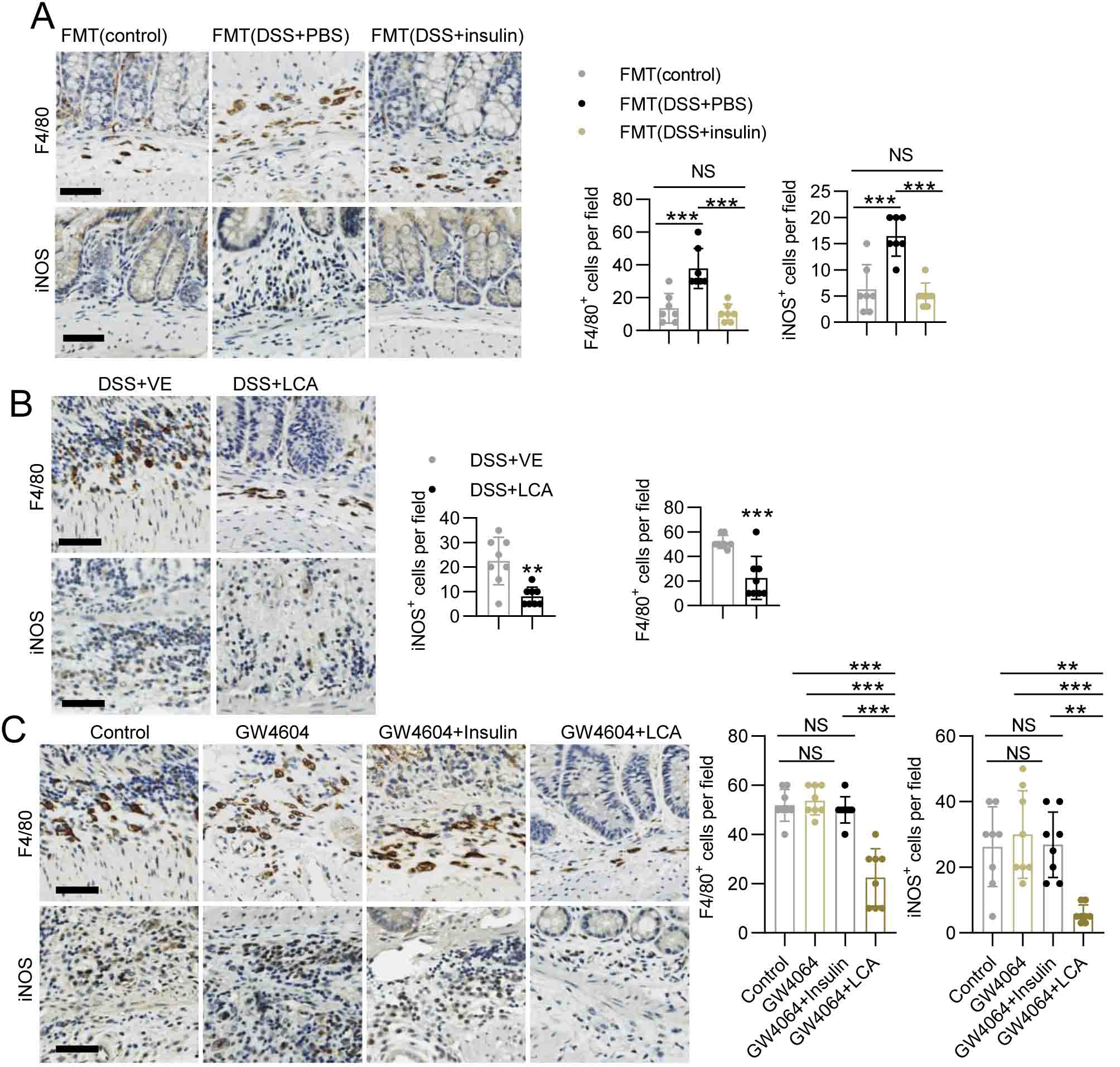


**Supplementary Figure 10.** Insulin inhibited M1 macrophage polarization through the LCA-Tgr5 pathway. **(A)** WT mice were treated with Abx for 5 days, and the mice were administered 2.5% DSS and underwent FMT of feces originating from the normal group, insulin-treated DSS group and PBS-treated DSS group. Representative images of F4/80 and iNOS immunohistochemical staining in sections of colon tissues. Scale bar=50 μm. **(B)** Mice were given water supplemented with 2.5% DSS and treated with 100 µL of a suspension of bile acid (5 mg of LCA) or vehicle control (VE) via the rectum on days 3, 5, and 7. Representative images of F4/80 and iNOS immunohistochemical staining in sections of colon tissues. Scale bar=50 μm. **(C)** GW4064 was orally administered at a dose of 10 mg/kg twice with a 12 h interval between doses. Then, 2.5% DSS was administered, and the mice were treated with insulin and LCA. Representative images of F4/80 and iNOS immunohistochemical staining in sections of colon tissues. Scale bar=50 μm. The data represent the mean ± SD. NS, not signiﬁcant; **P<0.01; ***P<0.001.


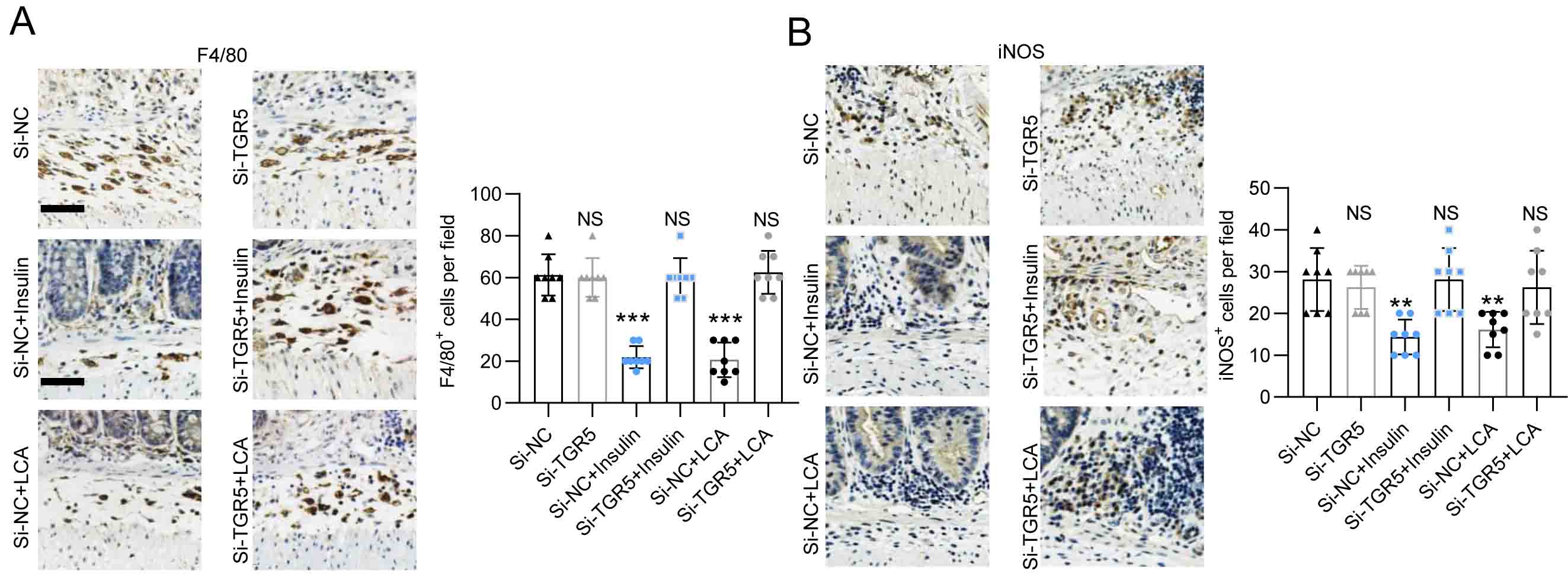


**Supplementary Figure 11.** LCA inhibited M1 macrophage polarization through TGR5. Mice were administered methylated siRNA targeting Tgr5 by intraperitoneal injection (1 OD/mouse) on days -3, 0 and 2 of the experiment. Three days after the first administration of siRNAs, 2.5% DSS was administered, and the mice were treated with insulin and LCA. Representative images of F4/80 **(A)** and iNOS **(B)** immunohistochemical staining in sections of colon tissues. Scale bar=50 μm. The data represent the mean ± SD. NS, not signiﬁcant; **P<0.01; ***P<0.001.
